# Reversible Adsorption and Interfacial Photoisomerization of Azobenzene Surfactants Studied by QCM

**DOI:** 10.1021/acs.langmuir.5c03617

**Published:** 2025-10-27

**Authors:** Maren Umlandt, Philipp Ortner, Nino Lomadze, Marek Bekir, Svetlana Santer, Yulia D. Gordievskaya

**Affiliations:** Institute of Physics and Astronomy, 26583 University of Potsdam, 14476 Potsdam, Germany

## Abstract

Photoresponsive surfactants offer a versatile approach for remotely controlling interfacial properties through light-triggered isomerization. Among them, azobenzene-based surfactants are particularly attractive due to their structural reversibility and stability under repeated irradiation. In this study, we investigate the dynamic adsorption and desorption behavior of the azobenzene-containing surfactant AzoC_6_ at a glass–water interface under controlled UV and blue-light illumination. Using quartz crystal microbalance (QCM) measurements, we show that the interfacial mass change is governed by the isomeric composition in the bulk solution: the *trans* isomer exhibits strong adsorption, while the *cis* isomer is significantly less surface-active. We further quantify the photoisomerization kinetics at the interface, revealing that the isomerization rate constant decreases with a lower *trans* isomer concentration due to a transition from a diffuse multilayer to a confined double-layer structure. At higher concentrations, the rapid exchange between *trans* and *cis* isomers sustains dynamic interfacial rearrangements, facilitating the formation of spatial isomer gradients. These gradients generate light-driven diffusio-osmotic flows, with a time evolution that reflects the interfacial photoresponse. Our findings provide mechanistic insight into light-induced interfacial processes and highlight the potential of azobenzene surfactants for designing stimuli-responsive systems and soft materials with remote, reversible control.

## Graphical abstract

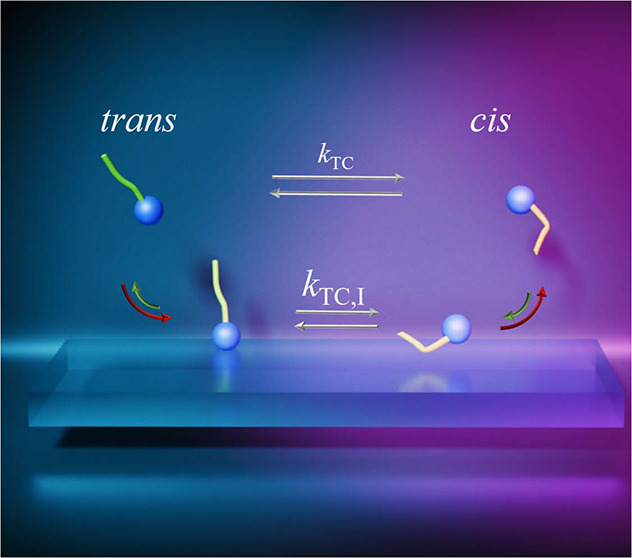

## Introduction

The design of smart materials relies on their ability to respond dynamically to external stimuli. Among various types of stimuli such as changes in ionic strength, pH, electric fields, or temperature^1–3^ light offers particularly attractive advantages. It can be applied remotely, with high spatial and temporal precision, and introduced into closed systems in a noninvasive and contactless manner.

Over the past decades, numerous light-responsive systems have been developed that encompass a wide range of applications. These include reversible nanoparticle precipitation and redispersion,^4,5^ photoresponsive polymer networks and microgels,^6,7^ surface relief gratings,^8,9^ light-guided fluid transport, manipulation of liquid crystals along planar interfaces,^10^ reversible wetting and dewetting of surfaces,^11^ emulsion destabilization,^12–14^ photoswitchable foams,^15,16^ controlled mixing of fluids,^17^ light-induced particle motion in quiescent liquids,^18^ selective separation of microparticles based on interfacial properties,^19,20^ and even biomimetic blood clotting using inorganic microparticles in microfluidic channels.^21^

One common approach to achieving light responsiveness in materials is the irreversible incorporation of photoactive molecular moieties into the chemical structure or matrix of the material itself.^22–24^ While effective, this strategy often involves complex synthesis procedures and limited adaptability, as any change in light-responsive behavior typically requires the design and synthesis of new compounds. An alternative and more versatile strategy is invasive triggering, in which inherently photoresponsive substances are introduced into a system to interact with the material and thereby transfer their light responsiveness. Photoresponsive surfactants are especially promising in this context, as they combine amphiphilic behavior with light-switchable properties within a single molecule. Their dynamic and often reversible interactions with interfaces allow light-triggered modulation of interfacial properties and, consequently, remote control over material behavior.^25–27^ As a result, such systems have attracted growing attention for their simplicity, adaptability, and potential in the development of responsive materials.^28–31^

Among the various classes of photoresponsive surfactants incorporating light-sensitive moieties such as stilbenes, spiropyrene,^32,33^ azopyrazole,^25^ azobenzene-based surfactants remain the most widely used. This is largely due to the favorable balance between long-term photostability and moderate quantum yields, resulting in minimal photodegradation over repeated switching cycles. As such, azobenzene-containing surfactants have found broad application in systems in which interfacial behavior can be modulated by light. When a solution of azobenzene-based surfactants is exposed to light, dynamic exchange processes between the bulk and interfacial regions (e.g., solid–liquid or air–liquid interfaces) are triggered. These exchanges are governed by the interplay of competing *trans* and *cis* isomers, both in solution and at the interface.^16,34,35^ For example, we previously demonstrated that the *trans* isomer of the surfactant 6-[4-(4-Hexylphenylazo)-phenoxy]-butyl-trimethylammonium bromide (C_4_-Azo-OC_6_-TAB, hereafter AzoC_6_; Figure 1a) exhibits significantly stronger adsorption at a glass–water interface compared to the *cis* isomer, with an adsorbed ratio of approximately 80:1 (*trans*:*cis*).^35^

**Figure 1:**
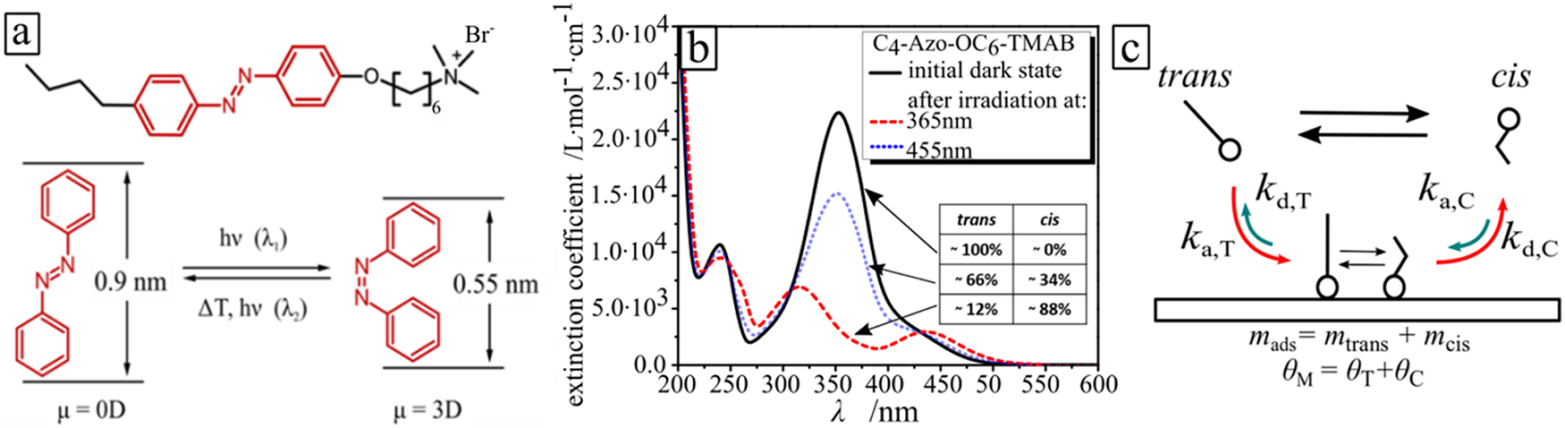
(a) Chemical structure of the photosensitive surfactant. The photoisomerization between *trans* and *cis* is depicted in the figure. (b) Adsorption spectra of the azobenzene-containing surfactant in the dark (black line), after exposure to UV (λ = 365 nm, red dashed line) and blue (λ = 455 nm, blue dotted line) light. (c) Scheme of the dynamic process of both isomers at the solid–liquid interface; the indexes of *k* correspond to the adsorption (a) or desorption (d) constants of the *trans* (T) and *cis* (C) isomers. *m* is the adsorbed mass of the *trans* and *cis* molecules on the surface. θ means the surface coverage resulting from the adsorbed mass.

These interfacial interactions were previously investigated by using ex situ measurements under static conditions. In these studies, the isomer ratio in solution was predefined by controlled bulk illumination, and no further light was applied to the interface during measurement. Under these conditions, we investigated the adsorption kinetics of AzoC_6_ and determined the adsorption and desorption rate constants for each isomer (Figure 1c).

However, the kinetics of photoisomerization and the corresponding rate constants for AzoC_6_ at the interface under in situ illumination remain unknown. In this study, we address this gap by analyzing the dynamic interfacial response under controlled UV and blue-light irradiation at varying bulk concentrations of the surfactant. From these dynamic measurements, we extracted the isomerization kinetics of AzoC_6_ at the glass–water interface.

The overall aim of this research is to gain a fundamental understanding of mass transport and interfacial dynamics at solid–liquid boundaries in aqueous solutions of photoresponsive surfactants. These findings may also serve as a model system for more complex light-driven phenomena, such as diffusioosmosis^36^ and localized light-induced interfacial transport.^37^

## Experimental Section

### Materials

#### Light-Responsive Surfactant

Azobenzene-containing trimethylammonium bromide surfactant, full name 6-[4-(4-Hexylphenylazo)-phenoxy]-butyl-trimethylammonium bromide (C_4_-Azo-OC_6_-TAB, Figure 1a), is synthesized as described elsewhere.^38^ The surfactant consists of a spacer of six methylene groups between the positively charged trimethylammonium bromide headgroup and the azobenzene unit with a butyl tail attached. A stock aqueous solution (Milli-Q) of the surfactant is adjusted to a concentration of 10 mM and diluted to the required concentrations. The *trans* isomer has a characteristic absorption band (π–π* transition) with a maximum at 351 nm (Figure 1b). The spectrum of the *cis* isomer has two absorption bands with maxima at 313 nm (π–π* transition) and at 437 nm (n-π* transition). The lifetime of the *cis* isomers in aqueous solution stored in dark or under illumination with red light of λ = 600 nm is 48 h (at room temperature of 20 °C), while the photoisomerization from the *cis* to *trans* state under irradiation with blue light (λ = 455 nm) takes place within seconds (light intensity dependent), with a fraction of *trans* and *cis* isomers of 66% and 34% for 1 mM aqueous solution at the photostationary state, respectively (Figure 1b), we used the dark state as an idealized reference case of 100% *trans* isomers.^39^ Under UV illumination (λ = 365 nm) at the photostationary state, the surfactant molecules are predominantly in the cis state, with a fraction of 12% and 88% for 1 mM surfactant solution.^40^

A borosilicate glass sensor (QS-QSX336, LOT Quantum Design GmbH) is used for the QCM-D measurements. Before each measurement, the sensor is cleaned according to a cleaning protocol from BiolinScientific. In short, the borosilicate glass sensors are placed for 10 min in the plasma cleaner (UV/Ozon ProCleaner, BioForce Nanoscience), followed by exposure to a bath with 2% sodium dodecyl sulfate (SDS) solution for 30 min and washing with Milli-Q water. Afterward, the sensors are dried in a nitrogen atmosphere and etched once more in a plasma cleaner for 10 min to eliminate the last possibly remaining residues.

#### Light Source

The samples are irradiated with a UV lamp of λ = 365 nm wavelength (M365L2 THORLABS Inc., Germany) and blue light of λ = 455 nm (M455L3C1 THORLABS Inc., Germany). For the in situ measurements, the intensities between *I* = 50 μW·cm^–2^ and 0.7 mW·cm^–2^ are applied to avoid the effect of light-induced detuning (LID).^41^

### Method

Quartz crystal microbalance with dissipation monitoring (QCM-D) measurements are performed on a four-chamber Q-Sense E4 instrument (BiolinScientific). The chamber is equipped with a window module, which allows irradiation of the sensor surface with light of different wavelengths in situ, i.e., during adsorption/desorption of the photosensitive surfactant. This enables us to observe the real-time adsorption kinetics under illumination.

Adsorption is monitored by collecting frequency and dissipation shift, Δ*f* and Δ*D*, from the third to the ninth overtone.^42^ Before each measurement, the resonance frequency of the crystal is determined in Milli-Q water. Values of Δ*f* and Δ*D* are recorded as long as the equilibrium of the signal is achieved.

Typical measurement is conducted as follows: first, Milli-Q water is pumped over the sensor to record a water baseline. After that, the liquid is replaced by the surfactant solution of the desired concentration, and the measurements are performed until saturation is reached. At this point, the pump is switched off, and the alternating irradiation with UV and blue light in both cases during the time needed to reach the saturation of the frequency and dissipation signal is conducted.

The flow rate is kept constant at 100 μL·min^–1^ for all measurements. When the solution is changed, the flow is stopped to prevent the formation of air bubbles in the tubing system. The temperature in the chamber is kept at 23 °C.

#### QCM Data Analysis

The adsorbed mass *m*_ads_ is determined by the modified Sauerbrey equation for unknown adsorbed films that considers so-called fluid effects to a first small load approximation:^43,44^

I $$m_{\mathrm{ads}}=-\frac{C}{n}\left(\Delta f+\frac{f_{0}\Delta D}{2}\right)$$

where *C* is the crystal constant (*C* ∼ 17.7 ng·cm^–2^), *n* is the overtone number, and *f*_0_ is the resonance frequency (*f*_0_ = 4.95 MHz). Because the data exhibit rather low dissipation, the mass is calculated from Δ*f* and Δ*D* values from the third to the ninth overtone after reaching a steady-state value and averaged over all overtone numbers. The surface coverage θ is calculated using the adsorbed mass *m*_*ads*_ obtained from the QCM-D measurement and the saturation mass *m*_sat_ corresponding to the complete surfactant adsorption (*m*_sat_ = 391.06 ± 7.88 ng·cm^–2^)^35^ according to the following relation:

II $$\theta =\frac{m_{\mathrm{ads}}}{m_{\mathrm{sat}}}$$

#### Theoretical Model of the Dynamic Exchange of Trans Isomers

Given that the surfactant exists in two isomeric forms, we consider a binary mixture of *trans* and *cis* isomers undergoing dynamic exchange at the solid–liquid interface. The overall surface coverage is therefore governed by the competitive adsorption of both isomer species. Consequentially, the total surface coverage, θ, is the sum of the surface coverages of *trans* (θ_T_) and *cis* isomers (θ_C_):

III $$\theta =\theta_{T}+\theta_{C}$$

Previously, we have shown that the surface coverage on a borosilicate glass surface is dominated by molecules in the *trans* state^35^ (see also short explanation in Supporting Information, Section 2), so the total surface coverage is simplified from θ = θ_T_ + θ_C_ to θ = θ_T_, and the Langmuir equation can be expressed as follows:

IV $$\theta_{T}=\frac{K_{T}^{e}c_{T}}{1+K_{T}^{e}c_{T}}$$

where $K_{T}^{e}=\frac{k_{a}}{k_{d}}$ is the equilibrium constant of the *trans* molecules, *k*_a_ and *k*_d_ are the rate constants of adsorption and desorption, respectively, and *c*_T_ is the bulk concentration of the *trans-* isomers.

The rate of the surface coverage by *trans* isomers can be expressed as follows:^45,46^

V $$\frac{d\theta_{T}}{dt}=-k_{\mathrm{TC},I}\theta_{T}I+k_{a}c_{T}\left(1-\theta_{T}\right)-k_{d}\theta_{T}$$

where *k*_TC,I_ is the photoisomerization rate constant from the *trans* to *cis* state at the interface and *I* is the intensity of irradiation. The first term of the equation, *k*_TC,I_θ_T_ *I*, describes the time-dependent change in the surface coverage of *trans* molecules θ_T_ due to the dynamic process during irradiation, *k*_a_ *c*_T_(1 – θ_T_), resulting from the change of *trans* isomers between surface and bulk. The rate constant of desorption of *trans* molecules from the surface is defined as *k*_d_θ_T_.

The solution of eq V (see Section 2, Supporting Information) describes the surface coverage after irradiation for the time period of (*t*_2_ – *t*_1_) with coverage θ_T,1_ at *t*_1_:

VI $$\theta_{T,2}=\theta_{\mathrm{eq}}-\left(\theta_{\mathrm{eq}}-\theta_{T,1}\right)\exp\left[-\left(k_{a}c_{T}+k_{\mathrm{TC},I}I+k_{d}\right)\left(t_{2}-t_{1}\right)\right]$$

where the concentration, *c*_T_, of *trans* isomers in the bulk at any time *t* during irradiation is given^39^

VII $$c_{T}=c_{T,0}\cdot \frac{k_{CT}+k_{TC}\cdot \exp\left(-\left[k_{TC}+k_{CT}\right]\cdot I\cdot t\right)}{\left(k_{CT}+k_{TC}\right)}$$

where *k*_TC_ and *k*_CT_ are the photoisomerization rate constants from the *trans* to *cis* and *cis* to *trans* state in the bulk, respectively, and *c*_*T*,0_ is the surfactant concentration in the bulk at time *t* = 0. Using eq VI, one gets the photoisomerization rate constant *k*_TC,I_ on the surface from of the measured adsorption curves.

COMSOL Multiphysics 6.3 (COMSOL Inc., Burlington, MA) is used to simulate fluid flow, the surfactant concentration distribution, and their adsorption onto the sensor surface. The model is implemented in 3D using a fine, physics-controlled mesh consisting of 372,433 elements (for more details, see Section 3 and Figure S2, Supporting Information).

## Results and Discussion

The dynamic adsorption and desorption behavior of a photosensitive surfactant at the solid–liquid interface is investigated using a quartz crystal microbalance with dissipation monitoring (QCM-D), equipped with a window module to allow in situ measurements under controlled light illumination. A typical data are displayed in Figure 2, which shows the frequency (Δ*f*) and dissipation (Δ*D*) change of the sensor coated with a borosilicate glass during adsorption of a photosensitive surfactant (*c*_azo_= 0.3 mM) under illumination with light of two different wavelength, i.e., UV (λ = 365 nm, *I* = 0.2 mW·cm^–2^) and blue (λ = 455 nm, *I* = 0.5 mW·cm^–2^). The concentration of 0.3 mM was chosen as a representative example to demonstrate the experimental procedure; this concentration does not have a specific physical significance but was selected because it clearly illustrates the difference between UV- and blue-light irradiation. The same procedure applies to other concentrations, as well. The measurements start with pure water (region I in Figure 2) set as the baseline, followed by adding the surfactant predominantly in *trans* conformation (dark state) at the 10th minute of measurements (region II in Figure 2). After equilibration of the signal, the pumping of the flow is stopped, and the illumination with either UV (red line) or blue light (blue line) is switched on (region III in Figure 2). As can be seen from Figure 2 (see also scheme in Figure 2a), the trans isomers adsorb to the glass surface, causing a decrease in the frequency and dissipation change (region II), while irradiation and consequent photoisomerization from the *trans* to *cis* state force the surfactant to desorb. The frequency increase is more pronounced during UV irradiation (from −15 Hz up to −8 Hz) (Figure 2b), while blue-light irradiation alters it only slightly (Figure 2c).

**Figure 2:**
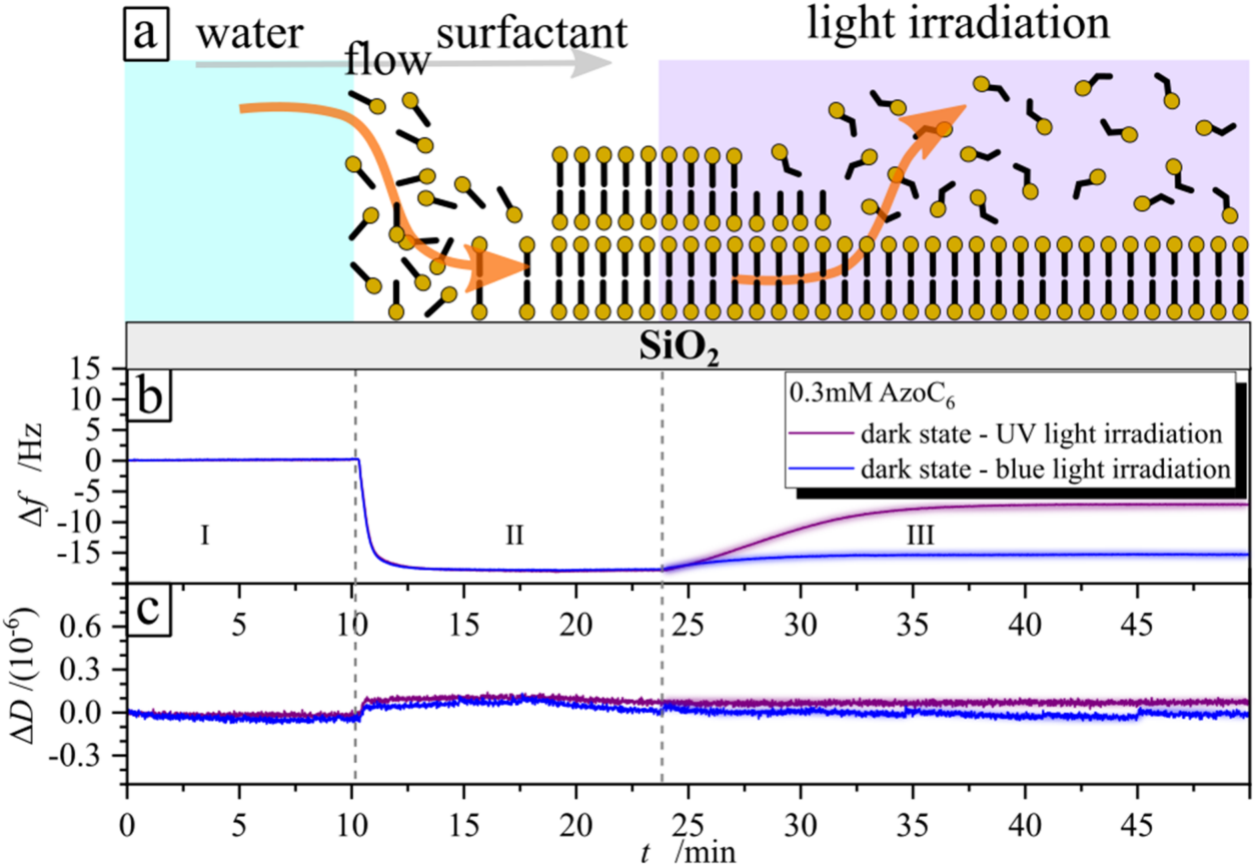
Results of QCM-D measurements during the adsorption/desorption of a photosensitive surfactant (*c* = 0.3 mM) on a borosilicate glass surface. (a) Scheme of surfactant adsorption and desorption on a surface. (b) Frequency shift Δ*f* and (c) dissipation shift Δ*D* as functions of time *t*. Region I indicates the water baseline, Region II corresponds to the adsorption of surfactant in the *trans* conformation, and Region III depicts the change in signal during exposure of the sample to UV (λ = 365 nm, *I* = 0.2 mW·cm^–2^, purple line) and blue light (λ = 455 nm, *I* = 0.5 mW·cm^–2^, blue line).

The adsorption and desorption behavior of *trans* isomers at the glass surface depends on the initial concentration of the surfactant solution (Figure 3). At lower concentrations, fewer molecules are available for adsorption, resulting in reduced surface coverage. Starting from a surfactant concentration of 0.5 mM (Figure 3c, dashed line), no further increase in the adsorbed amount is observed with increasing concentration, indicating that surface saturation has been reached.^35^ The COMSOL simulations of the adsorption of surfactants in the presence of flow accurately reproduce the experimental observations in Region II of the QCM experiment. The adsorption rate depends on the bulk concentration of the surfactants and the flow velocity of the liquid in the QCM chamber. In the current experiment, the volumetric flow rate is kept constant, V = 100 μL·min^–1^. Figure 4a shows the steady-state velocity distribution in the chamber, obtained using COMSOL. To calculate the adsorbed mass over time (Figure 4b), the experimentally obtained velocities, diffusion coefficient for the surfactant, *D* = 2.4 × 10^2^ μm^2^·s^–1^, and the Langmuir model for the adsorption are employed (Supporting Information for the details, Section 3). The circular region in the center marks the surface over which the adsorbed mass is averaged. As shown in Figure 4c, the equilibrium adsorbed mass increases with higher bulk concentration, and at higher concentrations, saturation is reached more rapidly.

**Figure 3:**
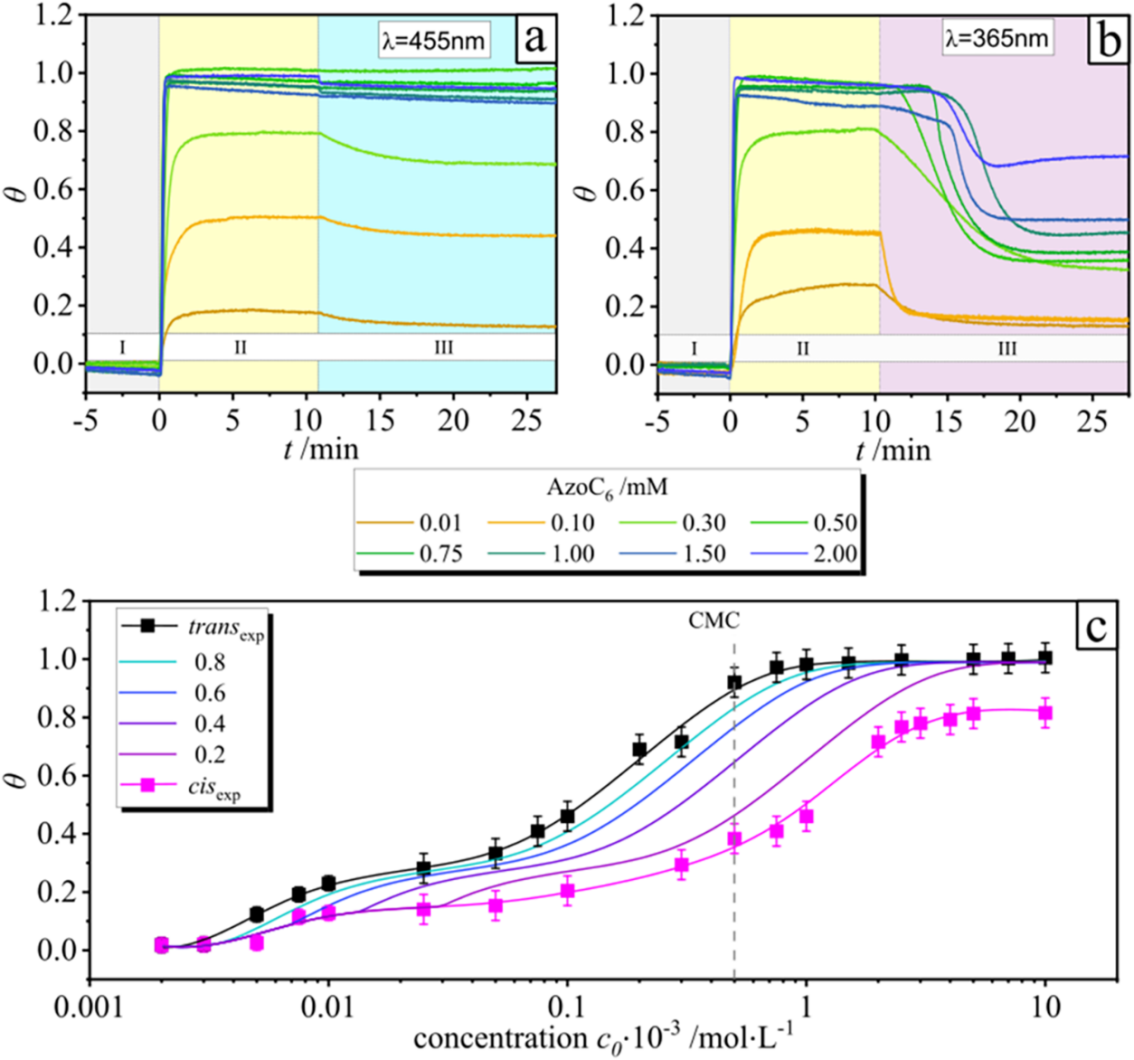
Surface coverage, θ, of the borosilicate glass surface as a function of time at different irradiations plotted for surfactant concentrations ranging between 0.01 and 2 mM (see the legend in the plots). Region I corresponds to the water baseline (a, b), Region II depicts surface coverage at different concentrations (a, b), and Region III shows surface coverage under irradiation with blue light (λ = 455 nm, *I* = 0.5 mW·cm^–2^) (a) and UV light (λ = 365 nm, *I* = 0.2 mW·cm^–2^) (b) for all surfactant concentrations, in situ measurements. (c) Adsorption isotherms of AzoC_6_ surfactants on a hydrophilic borosilicate surface under ex situ conditions. Black and pink lines show the dependence on the dark-state solution and the solution preirradiated with a UV LED (365 nm), respectively. The gradient lines describe the theoretical adsorption for different fractions of *trans* isomers in the solution.

**Figure 4:**
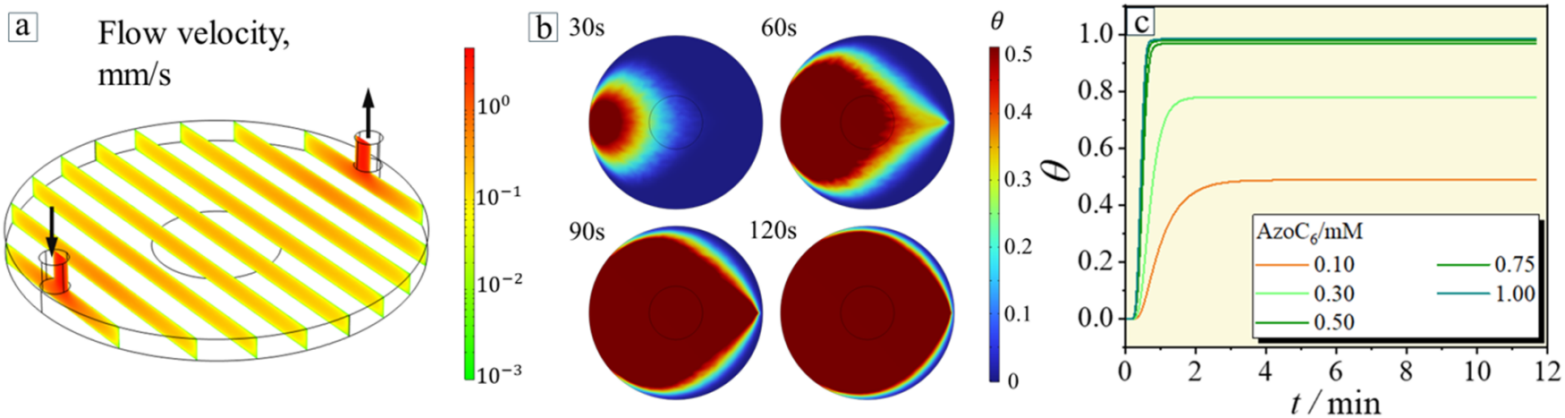
COMSOL simulation of the flow velocity of the liquid in the QCM chamber. (a) Steady-state flow velocity in the chamber at the volumetric flow rate of 100 μL·min^–1^. (b) Adsorbed surface coverage θ over time *t* for *c* = 0.1 mM (see also the corresponding Video S1). (c) Calculated dependencies of adsorbed surface coverage θ on time for different AzoC_6_ surfactant concentrations.

After the adsorption is complete, the sample is irradiated with light of different wavelengths. Upon light exposure (Region III, blue light (λ *=* 455 nm*)* in Figure 3a) and UV light (λ = 365 nm) in Figure 3b, the desorption of the molecules depends on concentration and wavelength. Indeed, in the case of the exposure to blue light at concentrations below the bulk CMC (*c*_CMC_ = 0.5 mM), a slight decrease in surface coverage is detected (Figure 3a), and irradiation of saturated surfaces (above the 0.5 mM) does not cause a detectable change in surface coverage (Figure S1). The situation is different for exposure to UV light. For the investigated concentration range of 0.01–2 mM, there is a pronounced decrease in adsorbed molecules during irradiation (Figures 3b and S1). At surfactant concentrations below the critical micelle concentration (CMC), the difference in surface coverage between the dark state and the UV-irradiated state increases progressively (Figure 3b). This difference reaches a maximum at the CMC (*c* = 0.5 mM) (Δθ_UV_ = 0.6). Beyond this point, the effect diminishes, and the difference in surface coverage decreases with further increasing concentration.

The desorption kinetics also exhibit a concentration-dependent behavior. Notably, at concentrations above the CMC, a delayed onset of desorption under UV irradiation is observed. In contrast to sub-CMC concentrations, where a decrease in surface coverage begins immediately upon illumination, the onset of desorption above the CMC occurs only after a certain period of exposure (see the curves starting from *c* = 0.75 mM in Figure 3b). This phenomenon is addressed and interpreted within the framework of a physical model introduced in the following section.

As previous studies indicate that adsorption above 0.005 mM is mainly due to *trans* isomers,^35^ the total adsorbed mass can be assumed to depend on their fraction in solution. Figure 3c shows the adsorption isotherm constructed for different *trans* isomer fractions, based on the given assumption and using previously obtained data.^35^ This enables us to estimate the adsorbed mass as a function of the surfactant concentration and the *trans*-to-*cis* isomer ratio over time and to interpret the newly observed dependencies presented in Figures 4–6. At a fixed AzoC_6_ concentration in bulk, *c*_0_, irradiation reduces the *trans* isomer content, leading to measurable differences in the desorbed mass under UV and blue light at various bulk concentrations. When started with the preirradiated solution (either UV or blue light), the irradiation itself does not result in the change of the coverage as shown in Figure 5 for three different surfactant concentrations ((a) *c*_azo_ = 0.1 mM, (b) *c*_azo_ = 0.5 mM, and (c) *c*_azo_ = 1 mM).

**Figure 5:**
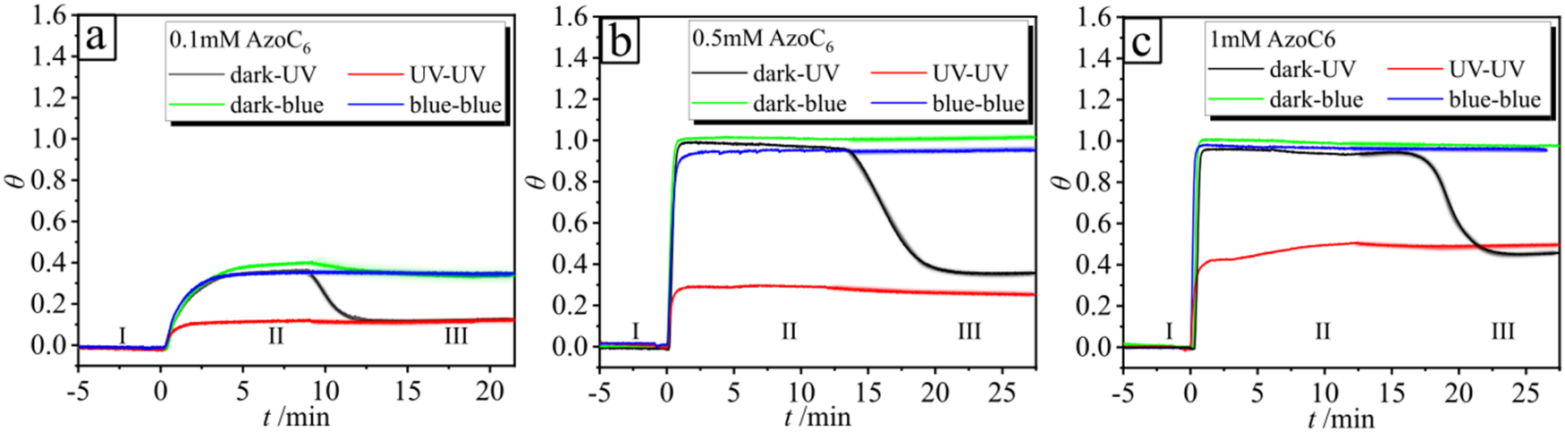
Surface coverage θ change as a function of irradiation wavelength over time *t* for different surfactant concentrations (a) 0.1 mM, (b) 0.5 mM, and (c) 1 mM AzoC_6_. Region I: water baseline. In Region II, two experiments are shown: the adsorption of isomers starts from the state when the majority of the molecules are in the *trans* conformation (black and green lines), followed by irradiation with UV and blue light (region III), respectively. The red line depicts the experiment where the initial state is a *cis*-enriched solution, followed by irradiation with UV light. The blue line depicts the situation where the solution is first preirradiated with blue light, and in Region III, the irradiation again with blue light is switched on. The intensities used are UV (λ = 365 nm) *I* = 0.2 mW·cm^–2^ and blue (λ = 455 nm) *I* = 0.5 mW·cm^–2^.

The adsorption–desorption process is reversible across all concentrations studied (Figure 6). However, at concentrations below 0.5 mM (the CMC), photoisomerization induced by alternating UV and blue light does not fully restore the original surface coverage observed in the dark state. For example, at 0.3 mM (Figure 6a), the surface coverage decreases from 0.75 to 0.3 (40% reduction) upon UV irradiation. Subsequent blue-light irradiation increases the coverage again, but the final value remains approximately 20% below that of the initial dark state.

**Figure 6:**
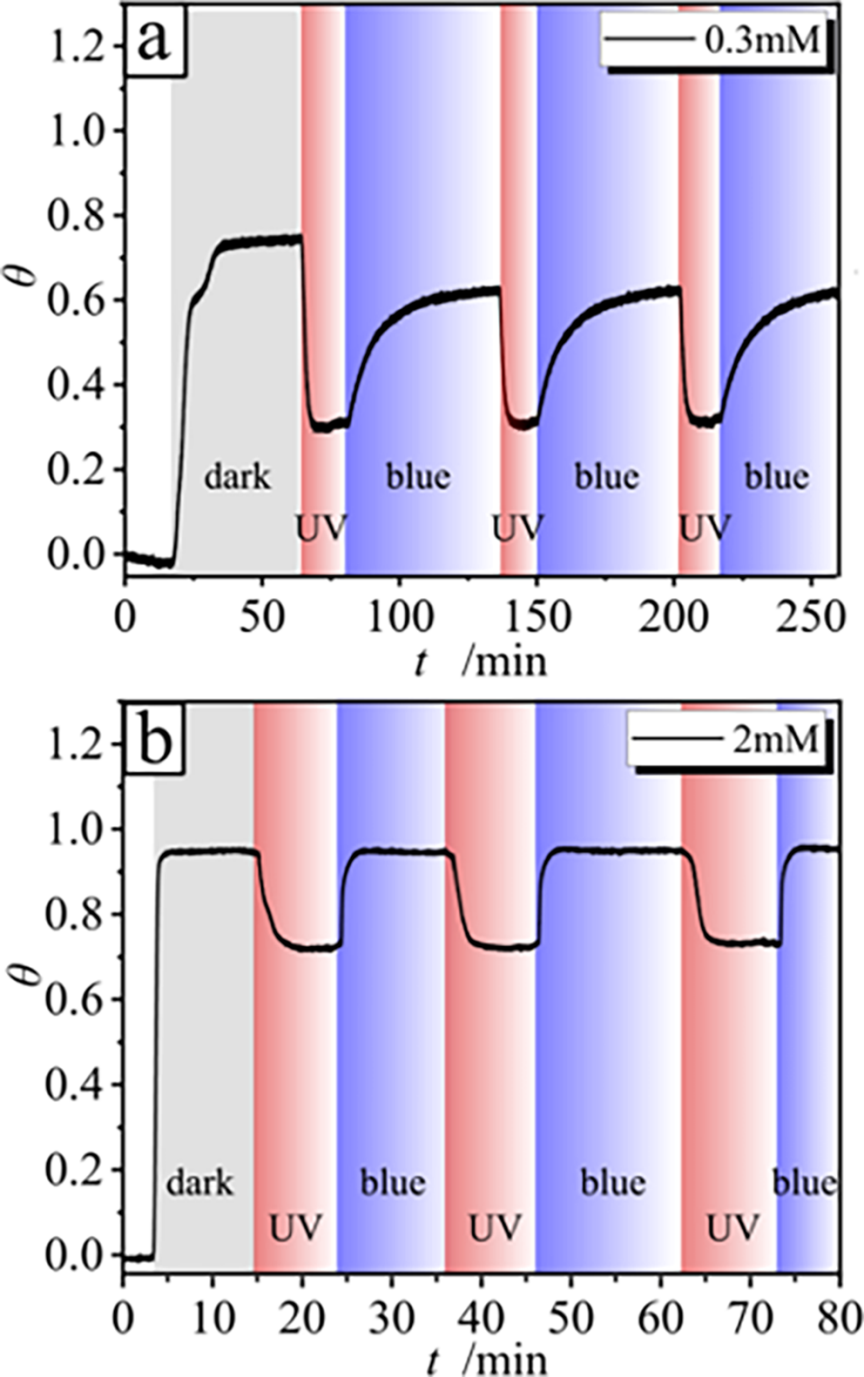
Shift of surface coverage θ over time *t* for (a) 0.3 mM and (b) 2 mM surfactant solutions during irradiation with the UV (λ = 365 nm, *I* = 0.2 mW·cm^–2^) and blue (λ = 455 nm, *I* = 0.5 mW·cm^–2^) light shown for 3 cycles.

At surfactant concentrations above the critical micelle concentration (CMC), the adsorption/desorption behavior exhibits a distinct pattern (Figure 6b). In the dark state, the surface is fully saturated with surfactant molecules. Exposure to UV light induces a reduction in surface coverage of approximately 25%, as observed at a concentration of 2 mM. Irradiation with blue light subsequently restores the surface coverage to its initial value. This reversible behavior can be attributed to the persistence of a fraction of *trans* isomers in the system following UV irradiation (Figure 3c). Specifically, after UV exposure, ca. 12% of the *trans* molecules remain in the system. At initial concentrations of 0.3 mM and 2 mM, this results in *trans* isomer concentrations of 0.04 and 0.24 mM, respectively, both of which are below the CMC. This reduction in *trans* isomer concentration contributes to the observed decrease in surface coverage. When irradiated with blue light, ∼66% of the *trans* isomers are converted back to their original state. For the 0.3 mM solution, this results in a *trans* isomer concentration of ∼0.2 mM, which remains below the CMC, preventing the surface from reaching full saturation. In contrast, at 2 mM, the *trans* isomer concentration rises to ca. 1.3 mM, a value well above the CMC, leading to complete surface saturation.

Further, it can be explained by considering the adsorption rate of only the remaining *trans* isomer in the solution during illumination with the blue and UV light. During illumination, the imbalance between two competing rates determines the surface coverage: the desorption rate of the *cis* isomer, which is proportional to the photoisomerization rate of the *trans* isomer at the interface, *k*_TC,I_, and the adsorption rate, *k*_a_, which is proportional to ∼*c*_T_ (1 – θ_T_) (see the Theory section for more details). Under blue-light illumination, a higher fraction of *trans* isomers remains in the solution (∼66%) compared to that under UV light (∼12%). Consequently, the adsorption rate is consistently higher under blue light than under UV irradiation, and this effect becomes more pronounced with an increasing bulk surfactant concentration.

At low concentrations, the lower availability of *trans* isomers limits adsorption. As a result, the surface coverage during illumination is reduced compared to the dark state, with a more significant decrease observed under UV light due to the lower *trans* isomer content in the photostationary state. In this regime, the surface coverage is adsorption rate-limited. In contrast, at higher surfactant concentrations, the rate imbalance shifts toward photoisomerization. The surface coverage during illumination remains high, following the same wavelength-dependent trend. Under blue light, the system clearly exceeds the CMC, leading to no significant change in surface coverage with or without illumination. In this regime, the surface coverage becomes limited by photoisomerization kinetics. Only minor differences are observed under UV light, consistent with its approximately 4-fold higher photoisomerization rate constant compared to blue light, *k*_TC,365 nm_ > *k*_TC,455 nm_ ∼ 0.03 > 0.008 cm^2^·(mW·s)^−1^.^35^

In other words, the dynamic exchange shifts the imbalance of adsorption (I) to the photoisomerization (II) rate-limited regime with increasing concentration from (I) to (II) relative to the applied wavelength.

### Kinetic Interpretation

To analyze the dynamic exchange kinetics, we assume that only *trans* isomers adsorb at the interface and contribute to the signal detected by the QCM-D technique. As discussed earlier, this has been experimentally confirmed: the *trans*-to-*cis* isomer ratio adsorbed at the interface is 80:1.^35^ During illumination, the concentration of trans isomers changes both at the interface and in the bulk solution. In the following analysis, we focus on the time interval during which the adsorbed mass undergoes measurable changes, as shown in the experimental data in Figure 7a (black line). Initially (time set to *t* = 0s), the surface of the borosilicate glass is saturated with molecules in the trans state. As a result of irradiation-induced isomerization from the *trans* to the *cis* state, the surface coverage decreases over time until a new equilibrium is reached. Further, we approximate the theoretical surface coverage using eq VI, and by setting parameters fixed to experimental conditions [*c*_0_ = 0.1 mM, *I* = 0.79 mW·cm^2^, *k*_TC_ of bulk is 0.05 cm^2^·(mW·s)^−1^]. The theoretical surface coverage is depicted as the red curve in Figure 7a, which shows that the photoisomerization rate constant at the interface, *k*_TC,I_, is 0.03 cm^2^·(mW·s)^−1^. This value is smaller in comparison to the bulk one (*k*_TC_ = 0.05 cm^2^·(mW·s)^−1^), *k*_TC_ > *k*_TC,I_ (see green curve in Figure 7a).

**Figure 7:**
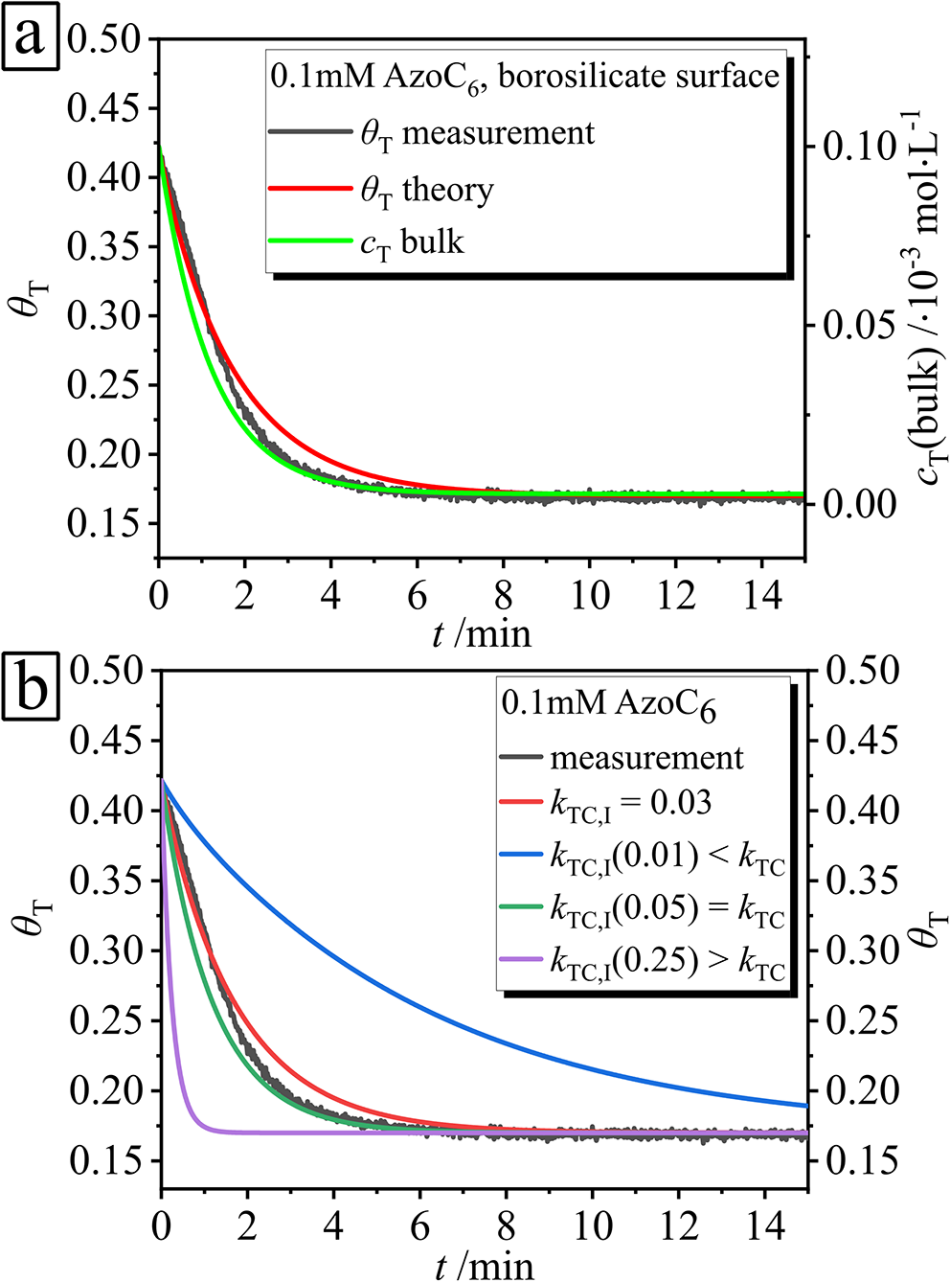
(a) Change of surface coverage θ_T_ over time for a 0.1 mM AzoC_6_ surfactant solution on a borosilicate surface. The black line shows the experimental data obtained during UV light irradiation (λ = 365 nm, *I* = 0^.2^mW·cm^–2^). The red line is the surface coverage calculated using eq VI. The green line depicts the change in the bulk concentration, *c*_T_, of the *trans* molecules as calculated using eq VIII. (b) Fitting of the surface coverage by different photoisomerization rate constant *k*_TC,I_ values.

To support this finding, the surface coverage data are fitted using different values of the interfacial photoisomerization rate constant, *k*_TC,I_, keeping all other parameters fixed (Figure 7b). When *k*_TC,I_ is set significantly lower than the bulk rate constant *k*_TC_ (blue curve), desorption is slower, resulting in a delayed and modest decrease in surface coverage. As *k*_TC,I_ increases (red and green curves), desorption accelerates, and a new equilibrium under UV irradiation is reached more rapidly. When *k*_TC,I_ > *k*_TC_ (violet curve), a sharp onset of desorption is observed, leading to a prompt decrease in surface coverage. These results indicate that to match the experimental data, *k*_TC,I_ must be smaller than the bulk rate constant at the given surfactant concentration.

As a next iteration, we fit the surface coverage change under illumination by varying the bulk surfactant concentration in the range between 0.05 and 2 mM.

During irradiation with blue light (λ = 455 nm, Figure 8a, blue dots and squares), the value for *k*_TC_ remains constant for all bulk surfactant concentrations below CMC. The same trend for *k*_TC_ is shown for UV light irradiation (λ = 365 nm, Figure 8a, purple dots and squares).

**Figure 8:**
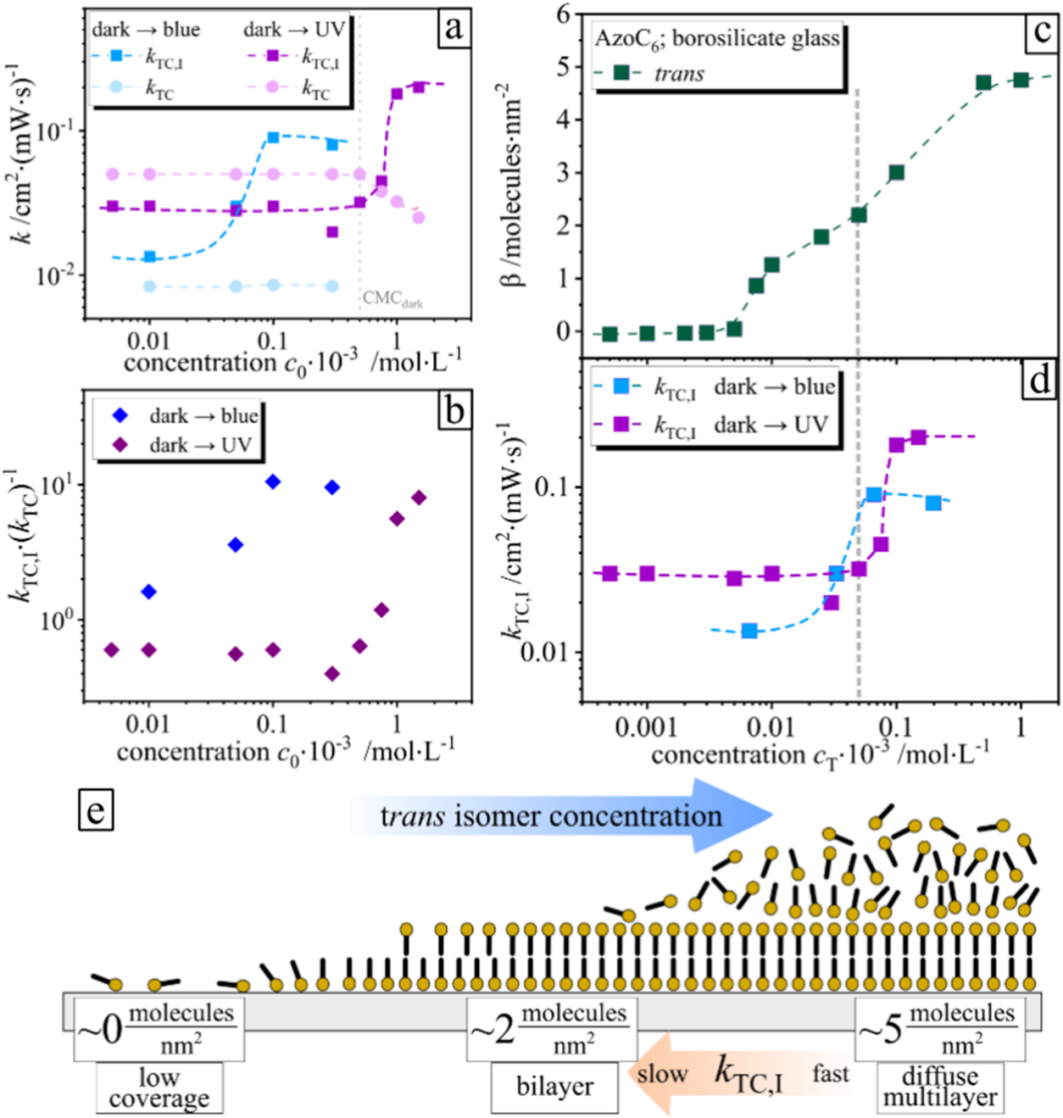
(a) Change of the photoisomerization rate constants as a function of surfactant concentrations for molecules in the bulk (*k*_TC_, blue and purple dots) and on the surface (*k*_TC,I_, blue and purple squares). (b) Comparison of rate constant ratios *k*_TC,I_·(*k*_TC_)^−1^ for different surfactant concentrations under exposure to blue (λ = 455 nm, blue squares) and UV (λ = 365 nm, purple squares) light. (c) Molecule density β as a function of *trans* surfactant concentration *c*_T_ in the *trans* state (dark state, green squares) on a borosilicate glass surface. (d) *k*_TC,I_ as a function of *trans* surfactant concentration irradiated with blue light (λ = 455 nm, blue squares) and irradiated with UV light (λ = 365 nm, purple squares). (e) Scheme of the process.

For both wavelengths, the value of *k*_TC,I_ shows the same trend as a function of the total surfactant concentration *c*_0_ = *c*_T_ + *c*_C_. The value of *k*_TC,I_ increases above a certain bulk concentration, which is *c*_0_ ∼ 0.03 mM for UV (λ = 365 nm) and *c*_0_ ∼ 0.4 mM for blue (λ = 455 nm) light. A similar trend is observed, although the initial concentration (*c*_0_) belongs to two different regimes. Since primarily *trans* isomers adsorb at the glass–water interface, the dynamic exchange during illumination is governed by the *trans* isomer concentration *c*_T_, rather than the total surfactant concentration, *c*_0_ = *c*_T_ + *c*_C_. During illumination, the value of *c*_T_ in the photostationary state changes as a function of applied wavelength and is *c*_T_ ∼ *c*_0_ 0.12 for UV (λ = 365 nm) and *c*_T_ ∼ *c*_0_ 0.66 for blue (λ = 455 nm) light illumination.^39^ With this correction, the onset of *k*_TC,I_ increase occurs at the same concentration threshold (*c*_T_ ∼ 0.04 mM) (Figure 8d). This increase correlates with structural surface coverage change of the adsorbed *trans* isomers beginning at *c*_T_ of 0.04 mM, where the surface undergoes a transition from a bilayer formation into a more diffuse multilayer-like formation (Figure 8e). A plot of the number of adsorbed molecules per nm^2^ (Figure 8c) reveals a clear transition in the adsorption behavior. At low concentrations, the surface coverage approaches ∼2 molecules·nm^–2^, consistent with the formation of a bilayer structure. At higher concentrations, however, the number of adsorbed molecules increases substantially beyond this value. Such high surface densities cannot be explained by a simple monolayer or bilayer arrangement and, therefore, strongly indicate the formation of additional adsorption layers (multilayers) at the interface.

In the following, a potential explanation for this phenomenon is explored. Molecules located within the bilayers are under strong confinement and are essentially locked in place. Furthermore, the benzene rings may undergo π–π stacking, leading to highly aligned molecular arrangements within the bilayers. Both factors can reduce the probability of the *trans*–*cis* photoisomerization, as this process requires lateral space for molecular rearrangement.^35^ Tightly aligned azobenzene molecules are therefore unable to switch to the *cis* form, a phenomenon confirmed by numerical studies on pure azobenzene molecules,^47–49^ as well as on azobenzene-based surfactants, in reasonable agreement with experimental findings.^39,50,51^ Upon exceeding a critical *trans*-isomer concentration, the adsorption probability at the solid–liquid interface increases, promoting the formation of a diffuse, less ordered secondary adsorption layer. This transition to a more diffuse interfacial structure at elevated concentrations is reflected by increased dissipation values recorded via QCM-D (Section 4 in the Supporting Information). The intricate dependence of dissipation on the surfactant concentration will be comprehensively analyzed in a subsequent study. In this sterically less constrained environment, the *trans*-to-*cis* photoisomerization rate is enhanced, resulting in an elevated effective photoisomerization rate constant, *k*_TC,I_ (Figure 8e). This behavior likely underpins the observed wavelength-independent trend, where the interfacial dynamics are predominantly controlled by the *trans*-isomer concentration, governing the isomeric exchange kinetics.

### Explanation of Desorption Delay: Model of Layered Bulk Concentration

The observed delay in surfactant desorption under ultraviolet (UV) irradiation represents a notable and nonintuitive characteristic of the system (Figure 3b). This phenomenon is absent under blue-light illumination. As a plausible explanation, a model is proposed that accounts for a layered concentration gradient of *trans* isomers within the bulk solution as well as the progressive penetration of UV light into deeper solution layers, leading to spatially heterogeneous photoisomerization dynamics. The Beer–Lambert law is commonly used to analyze the switching behavior of photosensitive compounds:

VIII $$I_{\mathrm{abs}}=I_{0}\left(1-10^{-{A\prime }^{\;}}\right)$$

where *I*_abs_ is the fraction of the initial light intensity *I*_0_. The parameter *A*′ is the absorbance by solution at the specified wavelength, and it is defined by the thickness of the sample *d*, as well as the extinction coefficients (Figure 1b) and concentrations of the *trans* and *cis* isomers, *c*_*T*_ and *c*_C_, respectively, as follows:

IX $$A^{\prime }=c_{T}\varepsilon_{T}d+c_{C}\varepsilon_{C}d$$

When the absorption coefficient is low (*A*′ < 0.1), the relationship between intensity and concentration remains linear.^52^ However, at high absorption coefficients, nonlinear effects arise; it occurs at high concentrations and/or large layer thicknesses. In this case, we propose to consider a solution of fixed concentration in the form of thin layers, within which the linear relationship is maintained (*I*_abs_·*I*_0_^–1^) < 0.2. Figure 9a shows the scheme, where the 0.5 mM AzoC_6_ solution is irradiated with a UV LED from the top, λ = 365 nm, *I* = 0.2 mW·cm^–2^. Rainbow-colored lines indicate considered layers, where the ratio between the concentration of *trans* isomers and absorbed light is linear. It can be observed that the concentration varies within each layer; it decreases more rapidly in layers near the top surface and experiences a delay in deeper layers (Figure 9b). This is due to the amount of incident light reaching each layer (Figure 9c). It turns out that the upper layers absorb the majority of the photons at the beginning of irradiation. Over time, the concentration of *cis* isomers in the system increases, the absorption parameter decreases by an order of magnitude (*ε*_T_^365 nm^ = 2.03 × 10^4^ L·mol^–1^ cm^–1^ and *ε*_C_^365 nm^ = 1.9 × 10^3^ L·mol^–1^ cm^–1^ in Figure 1b, these values correspond to rate constants in eq VII), and the layers begin to absorb an equal amount of photons. It is worth noting that the system is considered on time scales shorter than those required for mixing solely by diffusion, *t* < τ ∼ *H* ^2^·*D* ^–1^ ∼ 4200 s = 70 min, where *D* = 2.4 × 10^2^ μm^2^·s^–1^ is the diffusion coefficient for azobenzene surfactants^34^ and *H* = 1 mm is the height of the chamber. Thus, the observed delay in surfactant desorption under UV light irradiation can be explained by the physical picture presented above.

**Figure 9:**
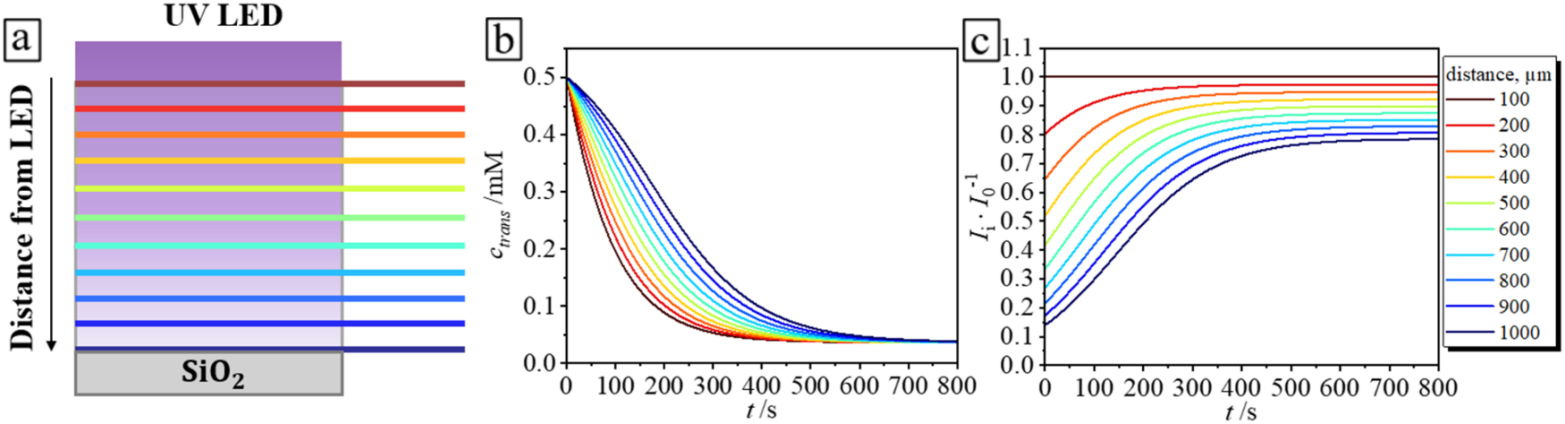
(a) Scheme of the irradiated bulk solution from the above with UV light. The rainbow color codec illustrates the considered finite layers at a specific distance from the light source. The time evolution of the concentration of *trans* isomers (b) and incident light (c) at different distances from the top surface over time in the case of λ = 365 nm and *I* = 0.2 mW·cm^–2^.

To gain further insight, we performed intensity-dependent measurements and the corresponding theoretical calculations for a solution with a surfactant concentration of *c*_azo_= 0.75 mM. The calculated concentration of *trans* isomers in the near-surface layer under irradiation at different intensities is shown in Figure 10a. Using the equilibrium adsorption isotherms from Figure 3c, the theoretical surface coverage was obtained and is presented in Figure 10b. A clear correlation is observed between the experimental surface coverage as a function of time and the theoretically calculated *trans* isomer concentration in the deeper layer, located 1 mm below the surface (the height of the QCM chamber). Notably, desorption occurs when the *trans* isomer concentration drops to approximately 0.6 mM, which is in agreement with the equilibrium adsorption isotherm. A comparison of the experimental and theoretical surface coverage curves confirms that the desorption delays under varying light intensities are consistent and the equilibrium values of θ are also in good agreement.

**Figure 10:**
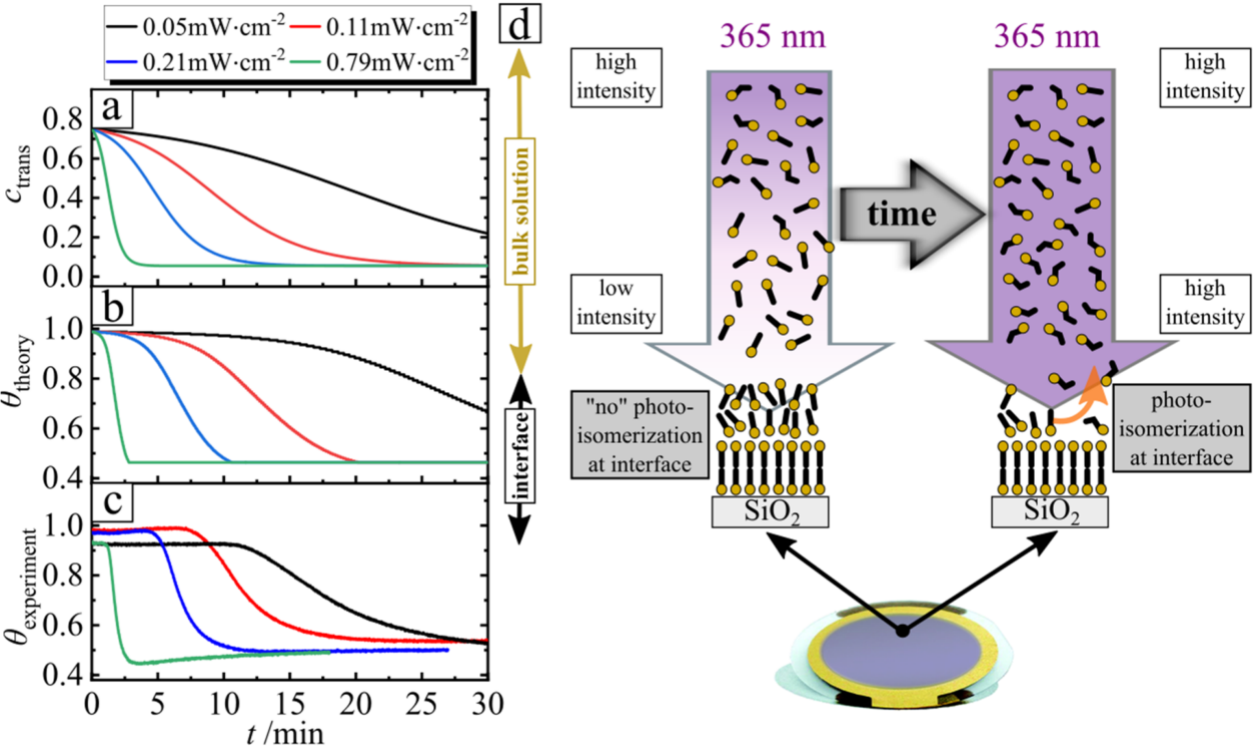
Desorption under the irradiation by UV LED with λ = 365 nm: (a) dependence of the concentration of *trans* isomers within the bottom layer; (b) theoretical θ_theory_ and (c) experimental θ_experimental_ surface coverage over time *t* in the case of different irradiation intensities *I*. The bulk surfactant concentration is *c*_azo_= 0.75 mM. (d) Scheme of the photoisomerization of surfactants in the bulk and on the surface, with the latter determining the number of adsorbed molecules over time.

Similar calculations for the system under irradiation with blue light are shown in Figures S3 and S4. Since the extinction coefficients (*ε*_T_^455 nm^ = 1.7 × 10^3^ L·mol^–1^ cm^–1^ and *ε*_C_^455 nm^ = 3.1 × 10^3^ L·mol^–1^ cm^–1^ in Figure 1b), and consequently absorption parameters, are smaller, there is no noticeable delay in the photoisomerization of molecules in the solution over the height under the current conditions. Moreover, using isotherm adsorption, we show that there is no significant desorption of molecules in the case of high initial concentrations. Returning to UV irradiation, it is important to note that the observed decrease in surface coverage in the initial moments of irradiation with high-intensity light is not an equilibrium effect, as the number of adsorbed surfactants eventually stabilizes at a constant value. This effect is of particular interest in future studies. We can speculate about possible causes, such as light interaction with the substrate, potential local heating, reorganization of surfactant molecular aggregates on the adsorption surface, and so on.

## Conclusion

In this study, we demonstrated that azobenzene-containing surfactants can be reversibly adsorbed to and desorbed from a glass–water interface under remote control by light irradiation; to reveal the processes involved, we performed in situ experiments under different wavelengths. The interfacial mass change is highly sensitive to the isomeric composition in the bulk solution: the *trans* isomer exhibits significantly higher surface affinity compared to the *cis* isomer, whose increased molecular polarity reduces its interfacial activity.^53^

Our experiments show that the light-controlled modulation of interfacial mass is most pronounced at low surfactant concentrations and under ultraviolet (UV) irradiation, whereas the effect is considerably diminished under blue light. This can be attributed to the dominant adsorption of the *trans* isomer; thus, the extent of mass change at the interface is governed by its absolute concentration. Under UV irradiation, the photostationary state favors the *cis* isomer, resulting in a low *trans* isomer concentration and, consequently, a strong desorption response. In contrast, blue light produces a nearly equal distribution of isomers, maintaining a relatively high *trans* isomer concentration that leads to nearly saturated interfacial coverage regardless of illumination.

To further elucidate the light-induced interfacial behavior, we investigated the photoisomerization kinetics at the interface by analyzing the temporal evolution of the interfacial mass during irradiation. The experimental data reveal that the photoisomerization rate constant decreases with decreasing *trans* isomer concentration in the bulk. This trend can be explained by a two-layer adsorption model: (i) a tightly packed, strongly adsorbed layer with highly aligned molecules and (ii) a more diffuse, loosely bound layer with less structural order. In the diffuse layer, molecular motion is less restricted, allowing efficient *trans*–*cis* isomerization. In contrast, steric hindrance in the densely packed layer suppresses isomerization, resulting in lower conversion rates.

Upon irradiation, the interface undergoes a structural transition from a diffuse multilayer to a more compact double-layer configuration as the limited *cis* isomer formation within the confined layer impedes further desorption-driven reorganization. Consequently, fast photoisomerization and dynamic interfacial exchange occur only at higher bulk surfactant concentrations (e.g., ≥1 mM), where rapid replenishment of the *trans* isomer sustains a multilayer adsorption regime.

This dynamic behavior enables the formation of spatial *cis* isomer concentration gradients near the interface driven by light. These gradients are sufficiently strong to induce repulsive diffusio-osmotic flows near microscopic objects. We demonstrate that the temporal evolution of these flows correlates with both the interfacial photoisomerization rate and the associated changes in interfacial mass. Furthermore, a delayed flow response under UV irradiation can be attributed to the high absorbance of the *trans* isomer at 365 nm, which attenuates the light intensity through the bulk before reaching the interface.

## Supplementary Material





## Notes

Video S1. Initial concentration evolution on the sensor when the azobenzene surfactant is added. (AVI)
Figure S1. Difference in surface coverage after irradiation for different surfactant concentrations. Figure S2. 3D geometry and mesh implemented in COMSOL. Figure S3. The concentration of trans isomers and incident blue light at different distances from the top surface over time. Figure S4. The concentration of trans isomers within the bottom layer over time under blue irradiation and the corresponding adsorption isotherms of trans isomers on a hydrophilic borosilicate surface. Figure S5. Frequency and dissipation shift for 0.3 and 2 mM AzoC_6_ surfactants (PDF)
