# Supplementary material for: Reversible Adsorption and Interfacial Photoisomerization of Azobenzene Surfactants Studied by QCM
Source: Langmuir. 2025 Oct 27;41(44):29567–77. doi: 10.1021/acs.langmuir.5c03617 (PMC12613797; doi:10.1021/acs.langmuir.5c03617)
Supplement: Supplementary file 2 [file la5c03617_si_002.pdf]

Supporting information

Reversible Adsorption and Interfacial Photoisomerization of Azobenzene Surfactants studied by QCM

Maren Umlandt, Philipp Ortner, Nino Lomadze, Marek Bekir, Svetlana Santer,* Yulia D. Gordievskaya*

Institute of Physics and Astronomy, University of Potsdam, 14476 Potsdam, Germany

Section 1. Langmuir model

The Langmuir model is used to describe the adsorption (r_a) and desorption (r_d) rates of surfactant:

$$\frac{d\theta}{dt} = r_a = k_a c(1-\theta) \tag{S1}$$

$$-\frac{d\theta}{dt} = r_\mathrm{d} = k_\mathrm{d}\theta \tag{S2}$$

with the surface coverage θ, the rate constants of adsorption k_a and desorption k_d and c_0 the surfactant concentration in the bulk.

Rearranged to express the equilibrium surface coverage, one obtains well-known Langmuir equation:

$$\theta_M = \frac{K_{TC}^{e} c_0}{1 + K_{TC}^{e} c_0} \tag{S3}$$

with the equilibrium constant $K_\mathrm{TC}^\mathrm{e} = \frac{k_\mathrm{a}}{k_\mathrm{d}}$ of the *trans* and *cis* molecules, which depends on the rate constant of adsorption k_a and desorption k_d and the bulk concentration c_0 of the *trans*- and *cis*- isomers ($c_0 = c_\mathrm{T} + c_\mathrm{C}$).

Section 2. Derivation of surface coverage for calculating photoisomerization rate constant $\boldsymbol{k_{TC,I}}$:

$\theta_M = \theta_T + \theta_C \qquad\qquad |\theta_T \gg \theta_C$

$\theta_{\mathrm{M}} = \theta_{\mathrm{T}}$

$$\frac{d\theta_T}{dt} = -\, k_{TC}\theta_T I + k_a c_T(1-\theta_T) - k_d\theta_T$$

$$= k_a c_T - k_a c_T\theta_T - k_{TC}\theta_T I - k_d\theta_T$$

$$= k_a c_T - (k_a c_T + k_{TC} I + k_d)\theta_T$$

Define: $\qquad k_a c_T = a;$

$$k_a c_T + k_{TC} I + k_d = b$$

$$\frac{d\theta_T}{dt} = a - b\theta_T$$

$$\int_{\theta_{T,1}}^{\theta_{T,2}} \frac{1}{a-b\theta_T}\, d\theta_T = \int_{t_1}^{t_2} dt$$

Substitute: $u = a - b\theta_T$; $du = -\, b\theta_T$

$$\int_{\theta_{T,1}}^{\theta_{T,2}} \frac{1}{a-b\theta_T}\, d\theta_T = -\frac{1}{b}\int \frac{1}{u}\, du = -\frac{1}{b} ln(u) + \mathrm{C}$$

Back substitute: $\qquad -\frac{1}{b} ln(a - b\theta_T)\Big|_{\theta_{T,1}}^{\theta_{T,2}} = t\big|_{t_1}^{t_2}$

$$-\frac{1}{b}\left[ln\left(a - b\theta_{T,2}\right) - ln(a - b\theta_{T,1})\right] = t_2 - t_1$$

$$ln\left(\frac{a-b\theta_{T,1}}{a-b\theta_{T,2}}\right) = b(t_2 - t_1)$$

$$\left(\frac{a-b\theta_{T,1}}{a-b\theta_{T,2}}\right) = e^{b(t_2-t_1)}$$

Resolve to $\theta_{T,2}$:

$$-\frac{1}{b}\left[ln(a - b\theta_{T,2}) - ln(a - b\theta_{T,1})\right] = t_2 - t_1$$

$$ln\left(\frac{a-b\theta_{T,2}}{a-b\theta_{T,1}}\right) = -b(t_2 - t_1)$$

$$\left(\frac{a-b\theta_{T,2}}{a-b\theta_{T,1}}\right)=e^{-b(t_2-t_1)}$$

$$-b\theta_{T,2}=\left(a-b\theta_{T,1}\right)e^{-b(t_2-t_1)}-a$$

$$\theta_{T,2}=-\frac{\left(a-b\theta_{T,1}\right)e^{-b(t_2-t_1)}-a}{b^2}$$

$$\theta_{T,2}=\frac{a}{b}-\frac{ae^{-b(t_2-t_1)}}{b}+\frac{b\theta_{T,1}e^{-b(t_2-t_1)}}{b} \qquad \left|\frac{a}{b}=\theta_{eq}\right.$$

$$\theta_{T,2}=\theta_{eq}-\theta_{eq}e^{-b(t_2-t_1)}+\theta_{T,1}e^{-b(t_2-t_1)}$$

$$\theta_{T,2}=\theta_{eq}+\left(-\theta_{eq}+\theta_{T,1}\right)e^{-b(t_2-t_1)}$$

$$\theta_{T,2}=\theta_{eq}+\left(-\theta_{eq}+\theta_{T,1}\right)e^{-(k_a c_T+k_{TC}I+k_d)(t_2-t_1)}$$

$$\theta_{T,2}=\theta_{eq}(1-e^{-b(t_2-t_1)})+\theta_{T,1}e^{-b(t_2-t_1)}$$

Units of the different constants:

K=[1/mol] : equilibrium constant

k_a=[1/(mol/s)] : adsorption rate constant

k_d=[1/s] : desorption rate constant

c_T=[mol] : bulk concentration of *trans* isomers

Preferential adsorption of *trans*-isomers on hydrophilic surfaces, $\theta_T \gg \theta_C$

As shown by Umlandt et al. (Langmuir,2021)[35], the adsorption of azobenzene containing surfactants at hydrophilic solid-liquid interfaces depends strongly on the molecular configuration. The *trans*-isomer, being more hydrophobic and rod-like, preferentially adsorbs to the hydrophilic silica surface. In this geometry, the polar head groups interact favorably with the hydrophilic substrate, while the elongated hydrophobic azobenzene units are stabilized at the interface. In contrast, the bent cis-isomer is more polar and better solvated in water which reduces its surface affinity. Consequently, adsorption layer at hydrophilic substrates are dominated by trans molecules and can be confirmed by the calculated equilibrium constant of *trans* and *cis* molecules ($K_T^e = 56088\frac{L}{mol}$, $K_C^e = 642\frac{L}{mol}$).

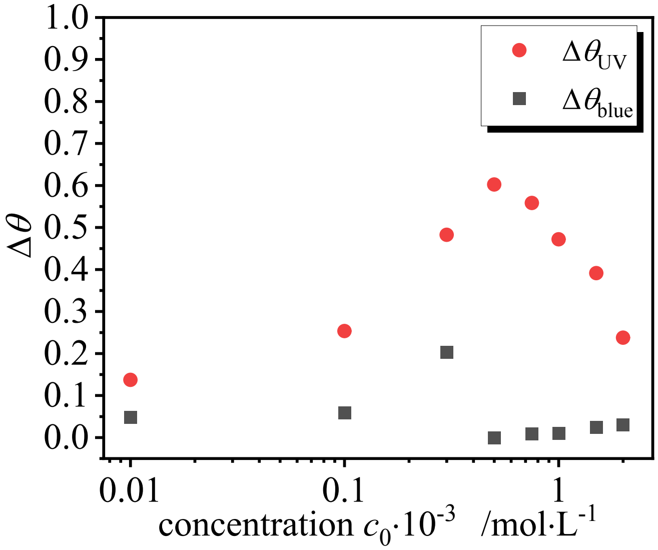

Figure S1. Difference in surface coverage $\Delta\theta$ after irradiation with UV and blue light for different surfactant concentrations. Red line is $\Delta\theta$ for the UV light and black line for blue light experiments.

Section 3. COMSOL Simulations

COMSOL Multiphysics 6.3 (COMSOL Inc., Burlington, MA) was used to simulate fluid flow, the surfactant concentration distribution, and their adsorption onto the sensor surface. The model was implemented in 3D using a fine, physics-controlled mesh consisting of 372,433 elements (**Figure 4a, Figure S2**). Flow within the system was modeled under steady-state laminar conditions with a fixed volumetric flow rate of V=100 μL/min. A no-slip boundary condition was applied to all walls. The diffusion of surfactant molecules and their adsorption onto the substrate surface were modeled using time-dependent simulations, employing the *Transport of Diluted Species* module for the bulk and the *General Form Boundary PDE* interface for surface reactions. The Langmuir model (**Eq. S1** and **Eq. S2**) was applied for the adsorption on the surface. We used the desorption constant $k_\mathrm{d} = 22 \cdot 10^{-3}\ s^{-1}$ for all concentrations under study. The adsorption constants are $k_\mathrm{a} = 210\ \mathrm{L}\ s^{-1} mol^{-1}$, $260\ \mathrm{L}\ s^{-1} mol^{-1}$ and $1400\ \mathrm{L}\ s^{-1} mol^{-1}$ for 0.1mM, 0.3mM and 0.5-1.0mM, respectively, to match the experimental data.

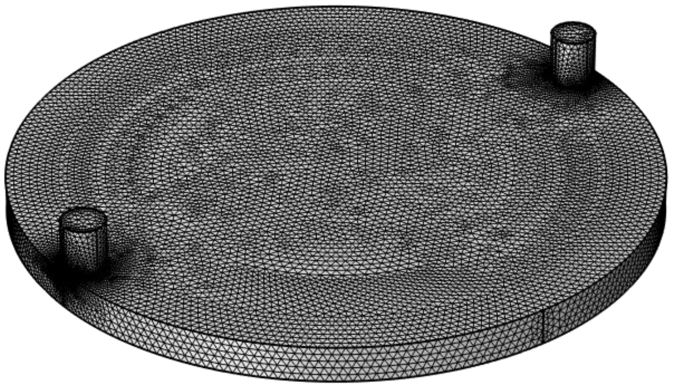

Figure S2. 3D geometry and mesh implemented in COMSOL.

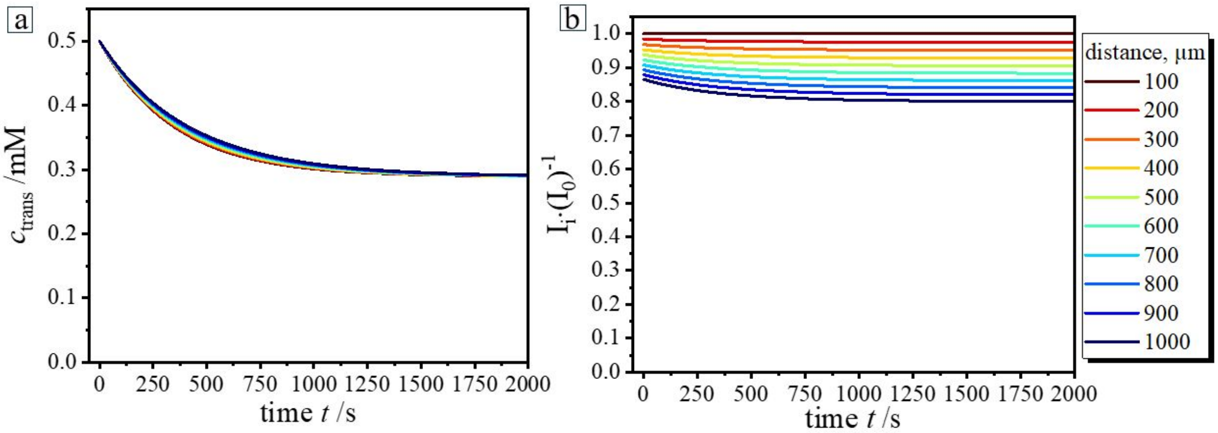

Figure S3. The concentration of *trans*-isomers (a) and incident light (b) at different distances from the top surface over time. c = 0.5mM, the intensity of blue light irradiation I = 0.2mW·cm^{-2}.

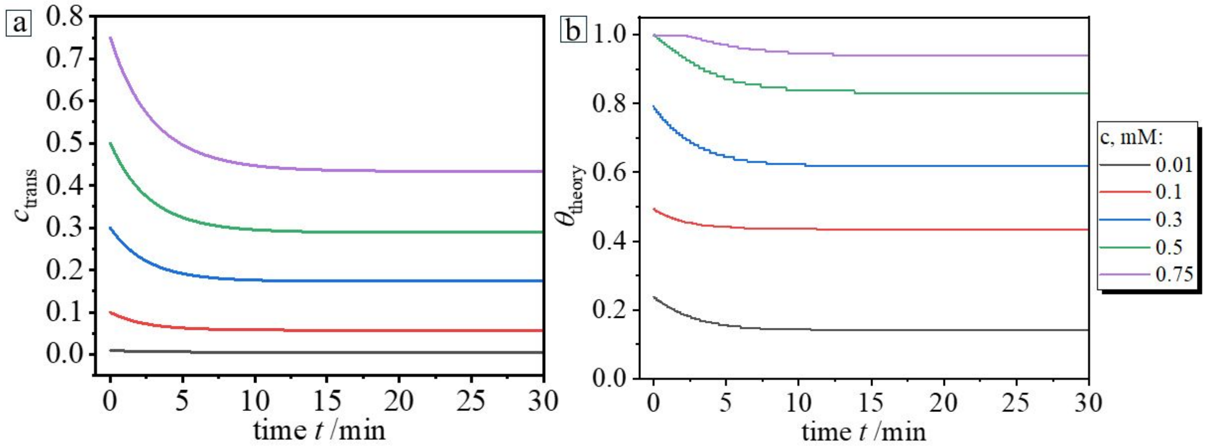

Figure S4. (a) The concentration of *trans*-isomers within bottom layer over time under blue irradiation with I = 0.5mW·cm^{-2} and (b) corresponding adsorption isotherms of *trans*-isomers on a hydrophilic borosilicate surface.

However, although the change in concentration is highest at c_{azo} =0.75 mM, the change in adsorbed mass is minimal and falls within the measurement uncertainty. The largest desorbed mass is observed at a concentration of c_{azo} =0.3 mM.

Section 4. Formation of multilayer/ diffuse layer

The dissipation shift observed during surfactant adsorption is significantly lower at a bulk concentration of 0.3 mM compared to 2 mM, indicating a pronounced difference in the mechanical properties of the resulting interfacial layers.

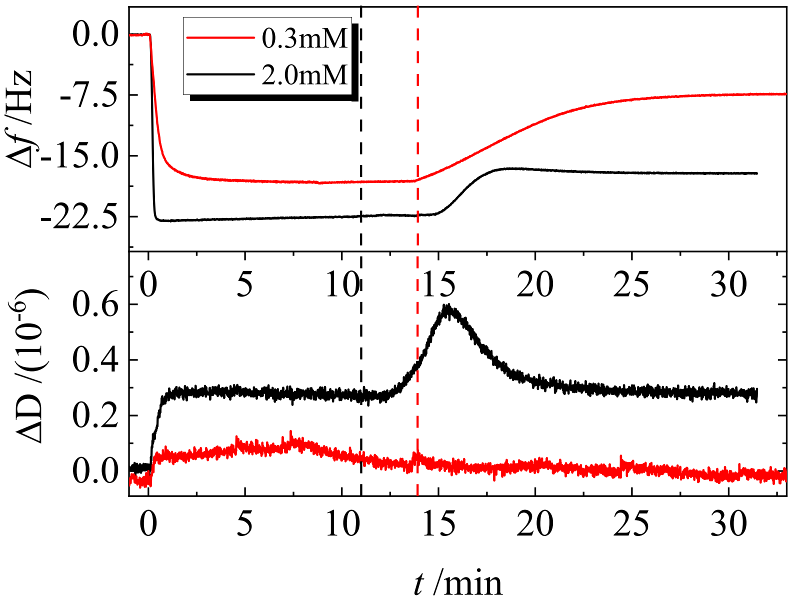

Figure S5. Frequency (top) and dissipation shift (bottom) for 0.3mM (red) and 2mM (black) $AzoC_6$ surfactant. t=0 indicate the start of adsorption of the surfactant molecules on the surface. The red and black dashed line indicate the start of UV light irradiation for 0.3mM and 2mM.

When interpreted alongside the corresponding frequency shift data, this suggests that the molecular architecture and packing of the adsorbed surfactant layer vary substantially with concentration. Specifically, a higher dissipation shift at 2 mM implies the formation of a more viscoelastic, loosely bound interfacial structure potentially consistent with the adsorption of surfactant aggregates such as multilayers or vesicular assemblies. In contrast, the lower dissipation response at 0.3 mM reflects a more rigid and compact adsorbed layer, likely corresponding to a monolayer or densely packed molecular arrangement. This interpretation is further supported by the calculated surface mass density and the estimated number of molecules per unit area. At elevated

concentrations (2–3 mM), the total adsorbed mass and associated dissipation behavior are consistent with the presence of multilayer structures or the non-specific adsorption of micellar/vesicular aggregates. Several previous studies have demonstrated that dissipation changes in QCM-D measurements provide critical insight into the viscoelastic properties and structural organization of adsorbed layers.[1−4]

1. Keller CA, Kasemo B. Surface specific kinetics of lipid vesicle adsorption measured with a quartz crystal microbalance. Biophys J. 1998 Sep;75(3):1397-1402. doi: 10.1016/S0006-3495(98)74057-3.
2. McCubbin GA, Praporski S, Piantavigna S, Knappe D, Hoffmann R, Bowie JH, Separovic F, Martin LL. QCM-D fingerprinting of membrane-active peptides. Eur Biophys J. 2011 Apr;40(4):437-46. doi: 10.1007/s00249-010-0652-5.
3. Dixon MC. Quartz crystal microbalance with dissipation monitoring: enabling real-time characterization of biological materials and their interactions. J Biomol Tech. 2008 Jul;19(3):151-8.
4. Rodahl, Michael, et al. "Simultaneous frequency and dissipation factor QCM measurements of biomolecular adsorption and cell adhesion." *Faraday discussions* 107 (1997): 229-246.

Section 5. Detailed data analysis and raw data

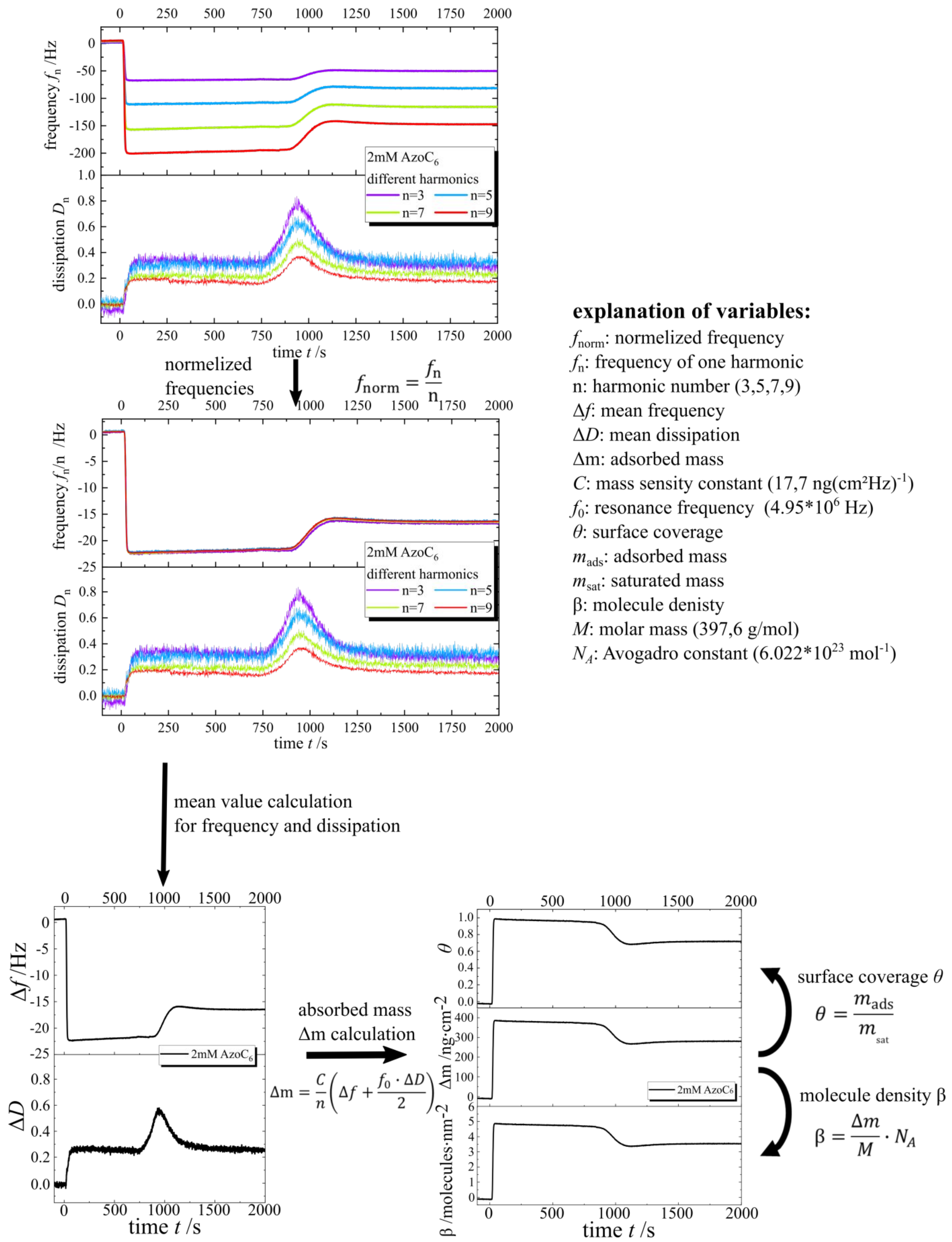

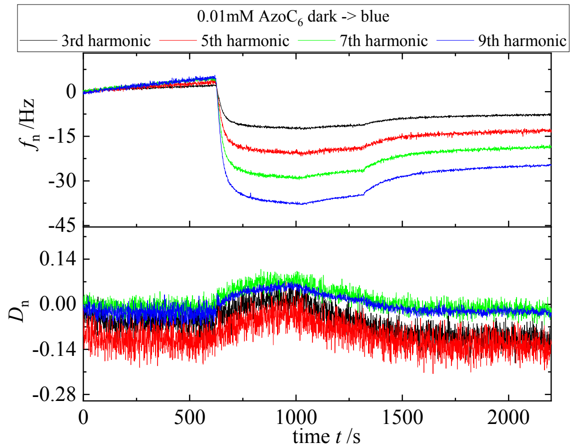
0.01mM AzoC6 dark -> blue
3rd harmonic 5th harmonic 7th harmonic 9th harmonic
fn /Hz
Dn
time t /s

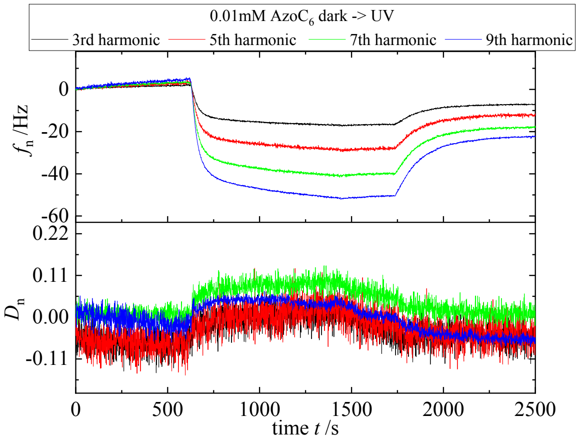
0.01mM AzoC6 dark -> UV
3rd harmonic 5th harmonic 7th harmonic 9th harmonic
fn /Hz
Dn
time t /s

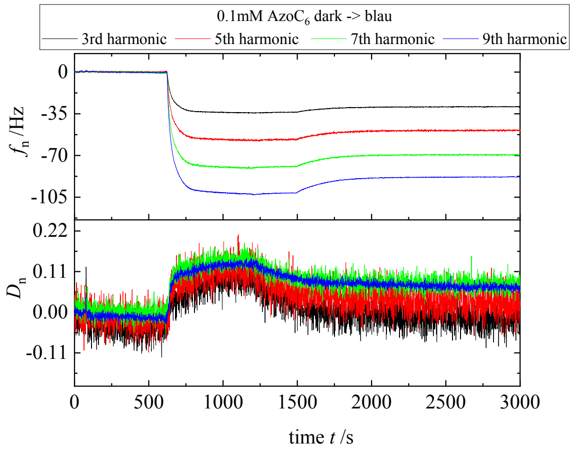
0.1mM AzoC6 dark -> blau
3rd harmonic 5th harmonic 7th harmonic 9th harmonic
fn /Hz
Dn
time t /s

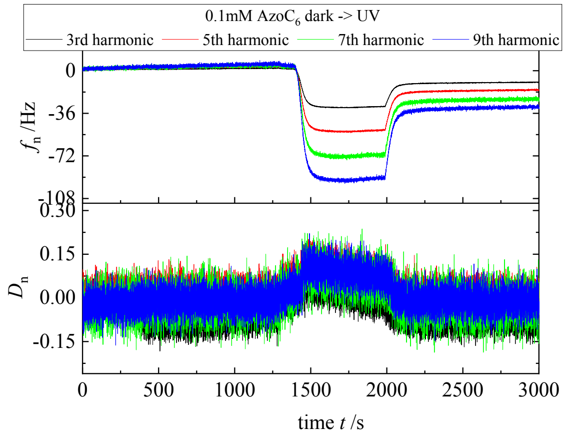
0.1mM AzoC6 dark -> UV
3rd harmonic 5th harmonic 7th harmonic 9th harmonic
fn /Hz
Dn
time t /s

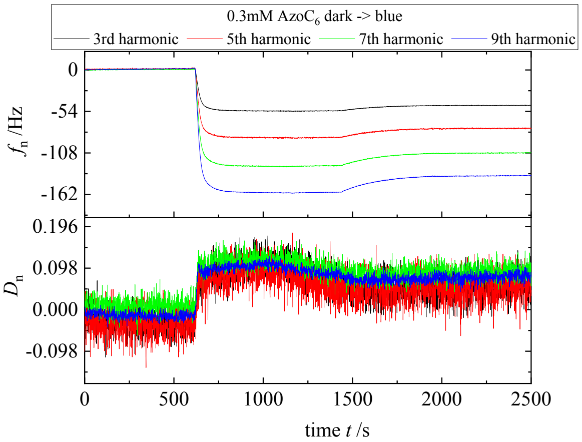
0.3mM AzoC6 dark -> blue
3rd harmonic 5th harmonic 7th harmonic 9th harmonic
fn /Hz
Dn
time t /s

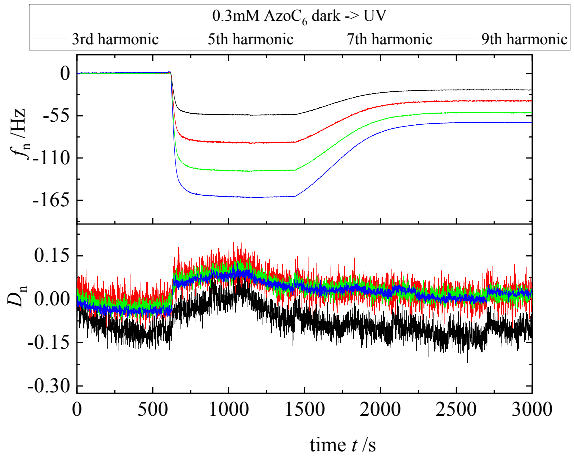
0.3mM AzoC6 dark -> UV
3rd harmonic 5th harmonic 7th harmonic 9th harmonic
fn /Hz
Dn
time t /s

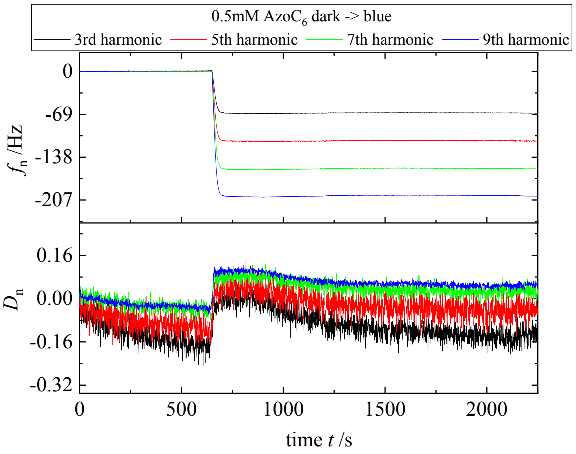
0.5mM AzoC6 dark -> blue
3rd harmonic
5th harmonic
7th harmonic
9th harmonic
f n /Hz
0
-69
-138
-207
D n
0.16
0.00
-0.16
-0.32
0
500
1000
1500
2000
time t /s

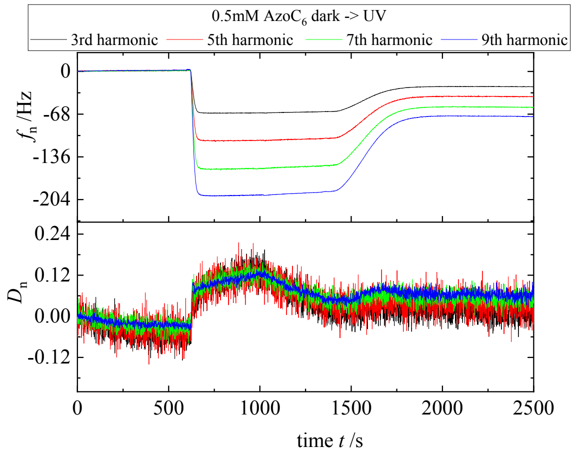
0.5mM AzoC6 dark -> UV
3rd harmonic
5th harmonic
7th harmonic
9th harmonic
f n /Hz
0
-68
-136
-204
D n
0.24
0.12
0.00
-0.12
0
500
1000
1500
2000
2500
time t /s

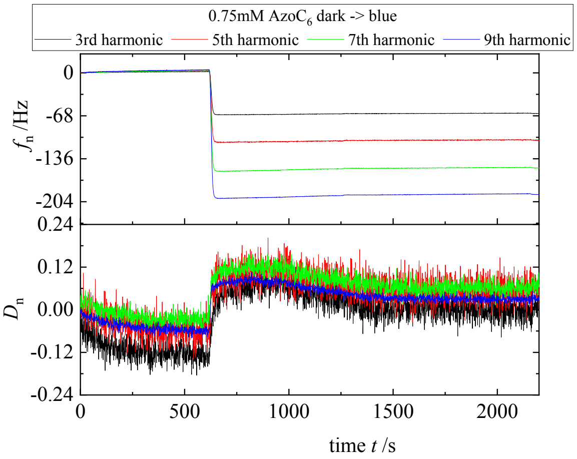
0.75mM AzoC6 dark -> blue
3rd harmonic
5th harmonic
7th harmonic
9th harmonic
f n /Hz
0
-68
-136
-204
D n
0.24
0.12
0.00
-0.12
-0.24
0
500
1000
1500
2000
time t /s

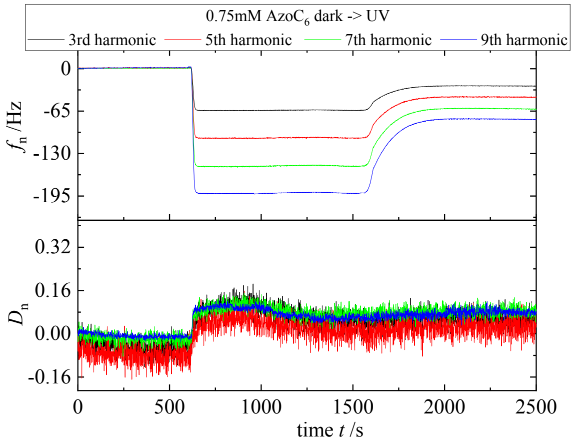
0.75mM AzoC6 dark -> UV
3rd harmonic
5th harmonic
7th harmonic
9th harmonic
f n /Hz
0
-65
-130
-195
D n
0.32
0.16
0.00
-0.16
0
500
1000
1500
2000
2500
time t /s

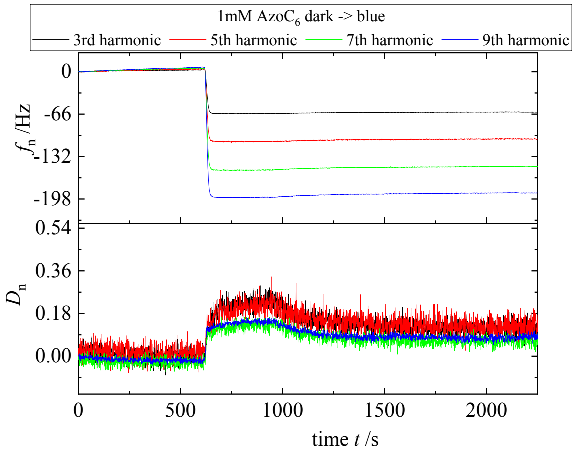
1mM AzoC6 dark -> blue
3rd harmonic
5th harmonic
7th harmonic
9th harmonic
f n /Hz
0
-66
-132
-198
D n
0.54
0.36
0.18
0.00
0
500
1000
1500
2000
time t /s

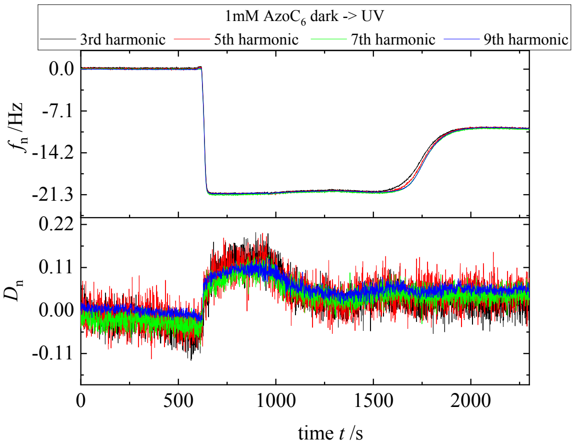
1mM AzoC6 dark -> UV
3rd harmonic
5th harmonic
7th harmonic
9th harmonic
f n /Hz
0.0
-7.1
-14.2
-21.3
D n
0.22
0.11
0.00
-0.11
0
500
1000
1500
2000
time t /s

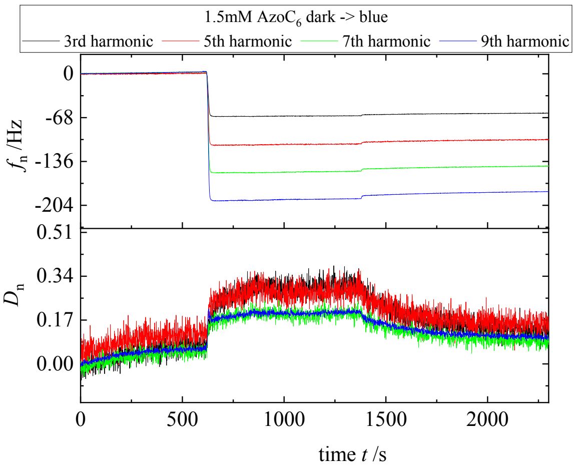
1.5mM $AzoC_6$ dark -> blue
3rd harmonic
5th harmonic
7th harmonic
9th harmonic
f_n /Hz
0
-68
-136
-204
D_n
0.51
0.34
0.17
0.00
0
500
1000
1500
2000
time t /s

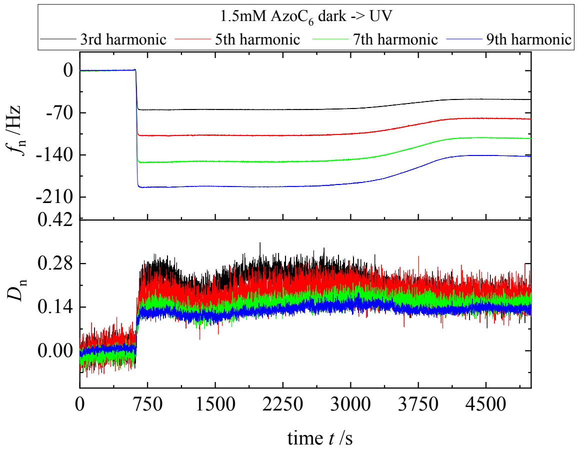
1.5mM $AzoC_6$ dark -> UV
3rd harmonic
5th harmonic
7th harmonic
9th harmonic
f_n /Hz
0
-70
-140
-210
D_n
0.42
0.28
0.14
0.00
0
750
1500
2250
3000
3750
4500
time t /s

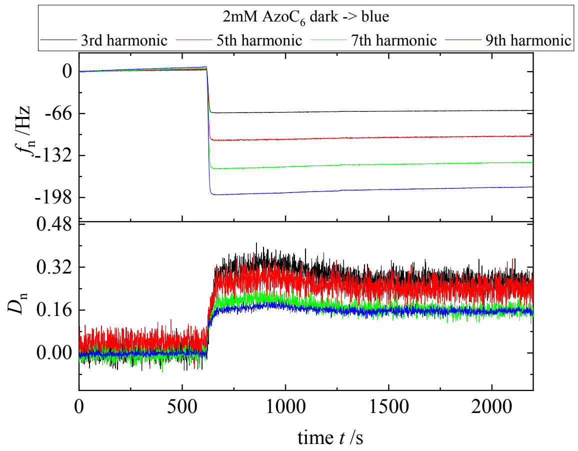
2mM $AzoC_6$ dark -> blue
3rd harmonic
5th harmonic
7th harmonic
9th harmonic
f_n /Hz
0
-66
-132
-198
D_n
0.48
0.32
0.16
0.00
0
500
1000
1500
2000
time t /s

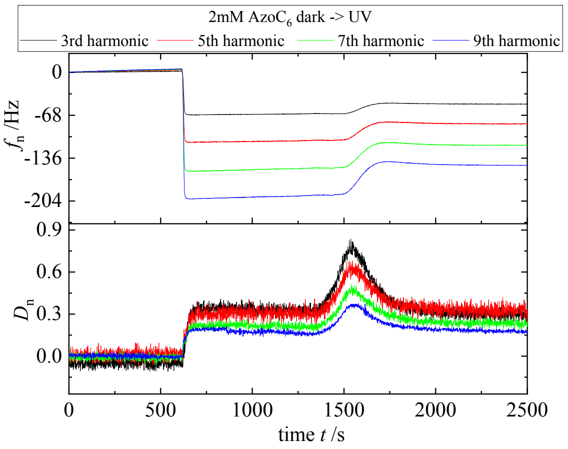
2mM $AzoC_6$ dark -> UV
3rd harmonic
5th harmonic
7th harmonic
9th harmonic
f_n /Hz
0
-68
-136
-204
D_n
0.9
0.6
0.3
0.0
0
500
1000
1500
2000
2500
time t /s
